# Edema and Structural Remodeling in Equine Suspensory Ligament Injury: A Severity Gradient Associated with Microvascular Dysfunction

**DOI:** 10.3390/ani16101432

**Published:** 2026-05-08

**Authors:** Constantin Lazăr, Vasile Vulpe, Andrei Radu Baisan, Dalma Pivariu, Florin-Dumitru Bora, Cristian Mihăiță Crecan

**Affiliations:** 1 Department of Clinics, Faculty of Veterinary Medicine, “Ion Ionescu de la Brad” Iași University of Life Sciences, 8 Mihail Sadoveanu Alley, 700489 Iași, Romania; constantin.lazar@iuls.ro (C.L.); vasile.vulpe@iuls.ro (V.V.); andrei.baisan@iuls.ro (A.R.B.); 2 Department of Toxicology, Faculty of Veterinary Medicine, University of Agricultural Sciences and Veterinary Medicine (UASVM) Cluj-Napoca, Mănăștur Street 3-5, 400372 Cluj-Napoca, Romania; dalma.pivariu@usamvcluj.ro; 3 Laboratory of Chromatography, Advanced Horticultural Research Institute of Transylvania, Faculty of Horticulture and Business for Rural Development, University of Agricultural Sciences and Veterinary Medicine of Cluj-Napoca, 400372 Cluj-Napoca, Romania; 4 Department of Anesthesiology and Surgery, Faculty of Veterinary Medicine, University of Agricultural Sciences and Veterinary Medicine (UASVM) Cluj-Napoca, Mănăștur Street 3-5, 400372 Cluj-Napoca, Romania; cristian.crecan@usamvcluj.ro

**Keywords:** suspensory ligament, equine lameness, microvascular dysfunction, edema, extracellular matrix remodeling, histopathology, magnetic resonance imaging, vascular remodeling, ligament injury

## Abstract

Suspensory ligament injury is a major cause of lameness in sport horses, yet its underlying mechanisms remain incompletely understood. The aim of this study was to characterize histopathological alterations of the equine suspensory ligament and to evaluate their association with imaging features. A comparative descriptive study was performed on sport horses with forelimb lameness, combining clinical evaluation, ultrasonography, magnetic resonance imaging, and histopathological analysis. Ligament samples were collected post-mortem from anatomically defined regions selected based on imaging findings to allow regional imaging–histopathology correlation. The results showed a consistent presence of edema in perivascular, interfascicular, and intrafascicular compartments, indicating a diffuse disturbance of tissue fluid balance. Lesions with lower severity were characterized by increased vascular permeability and mild extracellular matrix expansion, whereas lesions with greater structural severity demonstrated vascular remodeling, including endothelial thickening, vascular wall changes, and perivascular connective tissue proliferation, associated with collagen disorganization. Regional differences were observed, with more pronounced alterations in the proximal region compared to the branch region. These findings are consistent with a pattern of structural changes associated with microvascular alterations, demonstrating variation in lesion severity across samples and supporting an association between imaging categories and semi-quantitative histological features. However, given the cross-sectional design, causality cannot be established.

## Simple Summary

Lameness is a common problem in sport horses and can reduce both performance and welfare. One of the main causes is injury to the suspensory ligament, a structure that supports the limb during movement. Diagnosing these injuries can be challenging because standard imaging methods may not detect early tissue changes. In this study, we examined microscopic changes in the suspensory ligament and compared them with imaging findings. Tissue samples were collected after death from affected horses, from specific regions of the ligament identified using imaging, allowing direct comparison between structure and imaging appearance. Our findings suggest that small blood vessels may contribute to fluid accumulation within the tissue, particularly in less severe lesions. This fluid is associated with separation of collagen fibers and weakening of the ligament. In more severe lesions, both the vessels and surrounding tissue show more pronounced changes, including structural remodeling and disorganization. These results indicate that suspensory ligament injury follows a pattern related to lesion severity and is closely linked to changes in small blood vessels. Understanding these processes may help veterinarians detect injuries earlier and implement more targeted treatments, thereby improving recovery outcomes and performance after injury in sport horses.

## 1. Introduction

Suspensory ligament (SL) injuries are a major cause of lameness in horses and represent a significant clinical concern due to their impact on performance and their potential to limit or even end athletic careers across various equine disciplines [1,2,3,4]. The SL is anatomically divided into three regions: the proximal third (origin), the middle third (body), and the distal third, extending from the onset of branching to the insertion on the proximal sesamoid bones [5,6]. In sport horses, the proximal portion of the suspensory ligament is most commonly affected, with proximal suspensory desmitis occurring more frequently in the hind limbs. In contrast, body lesions are more commonly associated with racehorses, while branch injuries are observed across both racing and sport disciplines [7,8]. Lameness is one of the most prevalent clinical conditions in sport horses, significantly affecting both performance and welfare [9,10,11]. Suspensory ligament injuries represent a major contributing factor to lameness, with their anatomical distribution and clinical presentation varying between forelimbs and hind limbs [12]. This relationship highlights the functional interdependence between ligamentous and osseous structures within the suspensory apparatus [13]. Despite their clinical importance, accurate diagnosis remains challenging, as conventional imaging techniques may underestimate the extent of tissue alterations [14,15,16]. Advanced imaging modalities, particularly magnetic resonance imaging (MRI), have improved the detection of complex structural changes involving both the ligament and adjacent bone, including variations in signal intensity, the presence of intraligamentous edema, and bone marrow alterations, which may correlate with lesion severity [17].

The suspensory ligament is a highly specialized structure that is associated with stabilizing the metacarpophalangeal joint and optimizing locomotor efficiency [18]. Structurally, the suspensory ligament exhibits characteristics intermediately between tendon, ligament, and muscle tissue. It is predominantly composed of type I collagen organized into fascicles, supported by an extracellular matrix rich in proteoglycans [19,20,21]. This hierarchical organization enables efficient force transmission but is associated with limited regenerative capacity, making the ligament susceptible to microdamage associated with repetitive mechanical loading [22,23,24]. For clarity, the term ‘suspensory ligament’ is used consistently throughout this study to refer to this structure.

Tissue remodeling in tendons and ligaments is governed by a complex interplay of mechanical and biological factors [25,26,27]. Compared to bone, these tissues have a reduced capacity for adaptation, particularly in adult individuals [28,29,30]. Repetitive mechanical stress may lead to extracellular matrix degradation, collagen fiber disorganization, and cumulative microinjury [31,32]. Histologically, these changes are characterized by separation of collagen fascicles, expansion of the extracellular matrix, and alterations within the interfascicular compartment, ultimately contributing to a decline in mechanical integrity [33,34].

In recent years, increasing attention has been directed toward the role of vascular factors in the pathogenesis of ligament injuries [35]. Earlier concepts proposed the existence of hypovascular regions predisposed to injury; however, more recent evidence indicates that the suspensory ligament possesses a relatively uniform microvascular network [36,37]. This suggests that lesion patterns cannot be explained solely by regional hypoperfusion [38,39,40]. Instead, microvascular dysfunction—characterized by increased vascular permeability and structural alterations of the vessel wall—may play a critical role in disrupting tissue homeostasis and promoting lesion development [41,42,43].

Tissue edema, frequently identified through both imaging and histopathological examination, represents a key manifestation of these processes [44,45]. It reflects an imbalance between fluid influx and drainage within the ligament and is often distributed across perivascular, interfascicular, and intrafascicular compartments [46]. This diffuse pattern suggests the involvement of microvascular dysfunction and extracellular matrix alterations in lesions of lower structural severity [47,48]. Importantly, such changes align with structural alterations reported in lower-severity lesions within the pathological spectrum [49,50].

**Figure 1:**
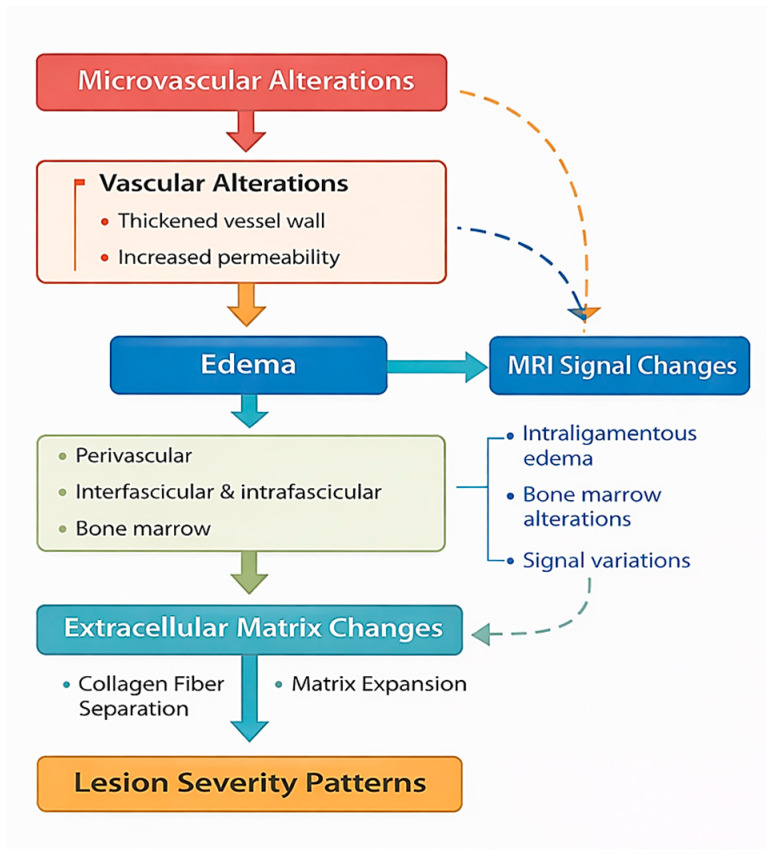
Conceptual schematic representation of the relationship between microvascular dysfunction, extracellular matrix remodeling, and lesion development in the equine suspensory ligament.

Despite the recognized clinical importance of suspensory ligament injuries, the relationship between microvascular alterations, extracellular matrix remodeling, and imaging findings remains incompletely understood [51,52]. In particular, the correlation between histopathological changes and imaging features requires further investigation to better elucidate the mechanisms underlying lesion severity and structural alterations [53,54]. To further illustrate the relationship between microvascular dysfunction, extracellular matrix remodeling, and lesion development, a conceptual schematic representation is provided (Figure 1).

The diagram illustrates the associations between microvascular alterations, edema, extracellular matrix changes, and ligament structural changes observed in this study. Microvascular alterations, including increased vascular permeability and structural changes of the vascular wall, contribute to the development of tissue edema. This edema is distributed across perivascular, interfascicular, and intrafascicular compartments and is associated with alterations in tissue fluid balance and structural changes within the ligament. These early alterations may represent functional changes associated with lower structural severity, in which the overall architecture of the ligament is still relatively preserved.

Fluid accumulation is associated with extracellular matrix disorganization, characterized by collagen fiber separation, loss of fascicular organization, and matrix expansion [42,45]. These alterations are accompanied by reduced tissue compactness and may be associated with compromised mechanical properties [19,24]. Vascular changes, including endothelial thickening and perivascular connective tissue proliferation, are also observed in affected samples and may contribute to tissue disorganization [4,52].

In parallel, these structural and fluid-related alterations are reflected in imaging findings, particularly magnetic resonance imaging (MRI), including signal intensity variations, intraligamentous edema, and bone marrow changes [8,16]. These imaging features provide a non-invasive representation of the underlying histopathological characteristics and may serve as indicators of lesion severity [15,51].

Together, these findings support an association between imaging characteristics and histopathological features, reflecting variability in lesion presentation rather than a defined sequence of lesion development [49,51].

The aim of the present study is to provide an integrated analysis of the histopathological and imaging alterations of the equine suspensory ligament, with particular emphasis on the association between microvascular features, extracellular matrix organization, and lesion severity. It is hypothesized that microvascular alterations are associated with fluid accumulation and edema, as well as with structural changes within the ligament [4,52]. By correlating histopathological findings with imaging features, this study seeks to improve the characterization of lesion-associated patterns and to support the development of more accurate diagnostic and clinical management strategies.

## 2. Materials and Methods

### 2.1. Ethical Approval

All procedures involving animals were conducted in accordance with relevant institutional and national guidelines for the care and use of animals, as well as in compliance with EU Directive 2010/63/EU on the protection of animals used for scientific purposes. The study protocol was reviewed and approved by the Research Ethics Committee of the “Ion Ionescu de la Brad” University of Life Sciences, Iași, Romania, under approval number ECA-00926, issued on 30 March 2026. Tissue samples were obtained post-mortem from 27 horses included in the study. No animals were euthanized specifically for research purposes; death or euthanasia occurred for reasons unrelated to this study. Informed consent was obtained from the owners of all animals included in the study.

### 2.2. Study Design

A total of 35 horses were initially evaluated for eligibility between January 2021 and December 2025, of which 8 were excluded based on predefined criteria, resulting in 27 horses included in the final study cohort. Tissue samples were obtained post-mortem from all included horses. Inclusion criteria comprised sport horses presenting with forelimb lameness and imaging-confirmed suspensory ligament lesions, with available clinical, imaging, and histopathological data (Table S1).

Based on predefined exclusion criteria, 8 cases were excluded due to incomplete imaging data, poor image quality, lack of clinical–imaging concordance, absence of MRI-detectable lesions, or concurrent musculoskeletal disorders (Table S2). All consecutive horses meeting the inclusion criteria during the study period were included, thereby minimizing the risk of selection bias.

For each case, clinical parameters (age, sex, breed, discipline, affected limb, AAEP lameness grade), duration of clinical signs (acute or chronic), and imaging findings were recorded. Histological samples were obtained from anatomically defined regions of the suspensory ligament (proximal region or branches), selected based on imaging findings to enable direct imaging–histopathology correlation.

Data were collected at predefined time points: at initial clinical presentation, signalment, affected limb, lameness severity (AAEP grade), and duration of clinical signs were recorded; ultrasonographic evaluation was performed prior to MRI as part of the clinical assessment; MRI findings were documented during the same clinical episode; and histological samples were obtained post-mortem from regions selected based on MRI findings to allow imaging–histopathology correlation.

### 2.3. Clinical Case Description

#### 2.3.1. Animal Characteristics

The study population included 27 sport horses (Table S1). The age of the horses ranged from 6 to 14 years. The study population included 8 stallions (M), 8 mares (F), and 11 geldings (G). Regarding breed distribution, 19 horses were Warmblood and 8 were Thoroughbred. Horses were equally distributed across disciplines, including dressage (*n* = 9), jumping (*n* = 9), and racing (*n* = 9). Forelimb involvement affected the left forelimb (LF) in 14 cases and the right forelimb (RF) in 13 cases. The severity of lameness ranged from AAEP grade 2/5 (*n* = 9) to 4/5 (*n* = 7), with grade 3/5 observed in 11 cases. Based on clinical history, 19 cases were classified as chronic and 8 as acute.

Regarding breed-related differences, the study population included both Warmblood and Thoroughbred horses. Within the present dataset, Thoroughbred horses were predominantly associated with acute presentations and more severe imaging findings, including partial or complete rupture, whereas Warmblood horses were more frequently associated with chronic cases and remodeling-type lesions. These observations reflect the distribution of cases included in the study and may be influenced by differences in use and discipline between breeds.

Regarding breed-related differences, Thoroughbred horses were significantly more frequently associated with acute presentations compared with Warmblood horses (*p* < 0.001). In contrast, Warmblood horses were significantly more commonly associated with chronic presentations and remodeling-type MRI lesions (*p* < 0.001). Thoroughbreds also showed a higher frequency of partial or complete rupture lesions (*p* < 0.001).

#### 2.3.2. Clinical History and Lameness Evaluation

Clinical history included duration of clinical signs, classified as acute (<6 weeks) or chronic (>6 weeks). Lameness severity was graded using the AAEP scale (0–5).

### 2.4. Standardized MRI Evaluation of Suspensory Ligament Pathology

MRI examinations were performed using a 0.27 T standing low-field system under sedation. A standardized multiplanar protocol included sagittal, transverse, and frontal planes. Sequences included T1W GRE, T2W FSE, T2*W GRE, and STIR FSE. T1W sequences were used for anatomical assessment, STIR for fluid and bone marrow edema detection, T2W for soft tissue evaluation, and T2*W for trabecular bone assessment. The MRI sequences and their diagnostic purposes are summarized in Table S3.

### 2.5. MRI Image Evaluation

MRI images were evaluated for osseous and soft tissue changes. Osseous structures, including the metacarpal/metatarsal condyles and the proximal phalanx, were assessed for signal intensity alterations. Bone marrow edema was defined as increased signal intensity on STIR sequences, whereas sclerosis was characterized by decreased signal intensity on T1W and T2W images. Soft tissue structures, including the suspensory ligament and its branches, were evaluated for size, structure, and signal intensity. Pathological findings included ligament thickening, loss of normal fiber architecture, and increased signal intensity on STIR and T2-weighted sequences. Synovial structures, including the metacarpo-/metatarso-phalangeal joint and the digital flexor tendon sheath, were assessed for effusion and synovial changes.

MRI images were independently evaluated by two experienced observers (a board-certified veterinary radiologist and a clinician with expertise in equine musculoskeletal imaging), both blinded to the histopathological findings. In cases of discrepancy, a consensus interpretation was reached following joint review.

### 2.6. Ultrasonographic Evaluation of the Suspensory Ligament

Ultrasonographic examinations were performed as part of the standard clinical assessment prior to magnetic resonance imaging (MRI) acquisition. Examinations were conducted using a high-resolution ultrasound system (GE Logiq E9 ultrasound system, GE Healthcare, Chicago, IL, USA) equipped with a linear transducer operating at 7.5–12 MHz. All images were acquired following a standardized scanning protocol. For the purposes of this study, ultrasonographic findings were retrospectively reviewed and compared with MRI features to evaluate imaging concordance.

#### Ultrasonographic Image Evaluation

Ultrasonographic images were assessed for echogenicity, fiber alignment, and overall structural organization of the suspensory ligament. Lesions were categorized into two primary patterns—focal and diffuse—based on echotexture and lesion distribution. Focal lesions were defined as localized hypoechoic or heterogeneous areas within the ligament, frequently associated with disruption of the normal fibrillar architecture. In contrast, diffuse lesions were characterized by more extensive alterations in echotexture, with reduced clarity of fiber alignment and the absence of a well-defined focal defect. Whenever possible, ultrasonographic findings were evaluated in comparison with the contralateral clinically normal limb at the corresponding anatomical level, in order to enhance the detection of subtle abnormalities. Image evaluation was performed by two experienced observers, blinded to MRI findings.

### 2.7. Tissue Sampling

Sampling was performed as soon as practically feasible after death, in accordance with clinical workflow. Limbs were maintained under refrigerated conditions (approximately 4 °C) prior to tissue collection. Although the exact post-mortem interval varied between cases, care was taken to minimize autolysis and post-mortem changes that could affect tissue morphology, particularly with respect to vascular features and fluid distribution. These precautions were considered especially important given the study focus on edema and vascular alterations.

Following collection, specimens were dissected to preserve anatomical orientation and structural integrity, and surrounding tissues were removed to isolate the ligament. Tissues were either fixed in 10% neutral buffered formalin for histological processing or temporarily stored at −20 °C prior to fixation. Before processing, samples were brought to room temperature.

#### 2.7.1. Sampling Regions

Samples were collected from predefined anatomical regions of the suspensory ligament, namely the proximal region (origin of the ligament) and the branch region, based on anatomical landmarks and imaging findings. For each case, lesion localization on MRI was determined according to its anatomical position within the ligament, taking into account its spatial relationship to adjacent osseous structures and ligament morphology.

During post-mortem dissection, these anatomical landmarks were used to guide targeted sampling of the corresponding regions identified on MRI. Care was taken to preserve spatial orientation and to maintain consistency between imaging planes and histological sectioning. Transverse sections were obtained in a standardized manner to approximate the imaging planes used during MRI acquisition.

Correlation between MRI and histology was based on anatomical and topographical matching; exact one-to-one spatial correspondence was not feasible. Particular attention was given to ensuring topographical correspondence between imaging findings and sampled tissue, allowing reliable regional imaging–histopathology correlation. Each horse contributed tissue from a single anatomical region only, selected based on MRI lesion localization.

#### 2.7.2. Sample Eligibility Criteria

Samples were excluded in cases where tissue integrity was compromised during collection or processing, or when significant concurrent musculoskeletal pathology affecting adjacent anatomical structures could interfere with interpretation.

### 2.8. Histological Processing

#### 2.8.1. Fixation and Embedding

Suspensory ligament samples were fixed in 10% neutral buffered formalin, followed by graded dehydration in ethanol, clearing in xylene, and paraffin embedding using a tissue processor (Leica TP1020, Leica Biosystems, Wetzlar, Germany).

#### 2.8.2. Sectioning

Paraffin-embedded suspensory ligament samples were sectioned at a thickness of 4–6 μm using a rotary microtome (Leica RM2235, Leica Biosystems, Germany). Sections were obtained according to the required anatomical orientation, including both transverse and longitudinal planes, depending on the region of interest. The sections were mounted onto glass slides (poly-L-lysine–coated slides) and dried to ensure proper adhesion. The prepared slides were subsequently used for routine histological staining and microscopic evaluation.

#### 2.8.3. Histological Staining

Sections were stained using Goldner’s trichrome according to standard histological protocols. This staining method was used to enhance the visualization of connective tissue architecture and vascular structures.

#### 2.8.4. Imaging–Histology Correlation

Imaging–histology correlation was performed by comparing ultrasonographic and MRI findings with corresponding histopathological features obtained from anatomically matched regions of the suspensory ligament. MRI signal characteristics (including STIR hyperintensity, T2 signal increase, and structural alterations) were systematically compared with histological parameters such as edema distribution, collagen organization, interfascicular spacing, and vascular changes.

The correlation analysis was performed on a per-case basis, integrating imaging and histopathological data to identify consistent structural and functional associations.

### 2.9. Histopathological Evaluation

Histopathological evaluation included extracellular matrix organization, collagen alignment, cellular morphology, vascular distribution, and edema. For each case, representative sections were analyzed, and multiple microscopic fields were evaluated to assign the semi-quantitative scores. All parameters were assessed on Goldner’s trichrome–stained sections using light microscopy (Olympus BX43, Olympus Corporation, Tokyo, Japan) at magnifications ranging from 4× to 40×. Evaluation criteria included fiber alignment, fascicular organization, interfascicular spacing, cellular density and morphology, and vascular features. All parameters were assessed comparatively between anatomical regions using a semi-quantitative scoring system. Semi-quantitative scores were assigned retrospectively based on predefined histopathological criteria. All slides were independently evaluated by two observers, and discrepancies were resolved by consensus to establish the final score. The evaluated histopathological parameters and their assessment criteria are summarized in Table S4.

For each case, 2–3 representative histological sections were evaluated, depending on tissue availability and quality. Within each section, 5–10 non-overlapping microscopic fields were systematically examined at different magnifications (4×–40×). Fields were selected to include both areas of maximal alteration and surrounding tissue, in order to avoid sampling bias and to capture overall lesion heterogeneity.

#### 2.9.1. Structural Organization

Structural organization included collagen alignment, fascicular architecture, interfascicular spacing, and matrix density. Parameters evaluated included degree of fiber disorganization, separation of collagen bundles, loss of fascicular definition, and changes in matrix density.

#### 2.9.2. Evaluation of Vascular Distribution

Vascular distribution was assessed based on the presence, density, and spatial organization of blood vessels. Vascular structures were assessed using Goldner’s trichrome staining.

#### 2.9.3. Edema Assessment

Edema was evaluated based on the presence of increased interfibrillar spaces, separation of collagen fibers, and expansion of the interfascicular matrix observed in Goldner’s trichrome–stained sections.

#### 2.9.4. Perivascular and Vascular Alterations

Perivascular and vascular alterations were assessed based on vessel morphology, including vessel size, wall thickness, and distribution, as well as the presence of perivascular connective tissue changes and inflammatory cell infiltration.

### 2.10. Comparative Analysis

#### 2.10.1. Branch Region vs. Proximal Region

Comparative analysis was performed between the proximal region and the branch region of the suspensory ligament to assess differences in histopathological parameters.

#### 2.10.2. Correlation of Imaging and Histopathological Findings

Imaging–histology correlation was assessed by comparing ultrasonographic and MRI features with corresponding histopathological changes, including echogenicity, signal intensity, structural integrity, collagen organization, interfascicular spacing, and edema.

### 2.11. Data Analysis

Data were analyzed using descriptive and non-parametric statistical methods, given the ordinal nature of the semi-quantitative scoring system and the absence of normality assumptions. Comparisons between anatomical regions (branch vs. proximal suspensory ligament) were performed using the Mann–Whitney U test for independent samples, as tissue samples were obtained from a single anatomical region per case. Differences between clinical severity groups (AAEP grades 2–3 vs. grade 4) were also assessed using the Mann–Whitney U test for independent samples. MRI findings were categorized into three ordinal groups reflecting lesion characteristics: category 1 (edema/hyperintensity), category 2 (remodeling/fibrosis), and category 3 (partial or complete rupture), based on signal intensity and structural alterations observed on MRI.

Correlations between MRI findings and histopathological scores were evaluated using Spearman’s rank correlation coefficient (ρ), with corresponding 95% confidence intervals calculated using established methods for non-parametric estimates. Interobserver agreement was assessed using Cohen’s kappa coefficient (κ), also reported with 95% confidence intervals. Data are presented as median and interquartile range (IQR) for ordinal variables, and as mean ± standard deviation (SD) for continuous variables, as appropriate. Given the relatively small sample size (*n* = 27) and the limited size of some subgroups, the study was designed as an exploratory analysis aimed at identifying biologically and clinically relevant patterns rather than providing confirmatory statistical inference. A two-tailed *p*-value < 0.05 was considered statistically significant. No formal correction for multiple comparisons was applied, as this would substantially increase the risk of type II error in the context of a limited dataset. Therefore, the reported *p*-values should be interpreted cautiously as descriptive indicators of association rather than definitive evidence.

Given the ordinal nature of the data and the relatively small sample size, no formal normality testing was performed, and non-parametric statistical methods were applied.

### 2.12. Semi-Quantitative Scoring and Observer Agreement

Histopathological parameters were assessed using a semi-quantitative scoring system defined as follows: 0 = absent, 1 = mild, 2 = moderate, and 3 = severe. Histopathological changes were evaluated using a semi-quantitative scoring system ranging from 0 to 3, based on structural, vascular, and extracellular matrix alterations (Table S5). Scoring was performed comparatively for proximal and branch regions based on predefined morphological criteria. A score of 0 indicated absence of detectable changes; 1 (mild) indicated minimal alterations, such as slight collagen fiber disorganization or mild interfascicular separation; 2 (moderate) indicated clearly visible structural changes, including evident fiber disruption, increased interfascicular spacing, or moderate cellular and vascular alterations; and 3 (severe) indicated marked structural disruption, including loss of normal fascicular architecture, pronounced edema, or extensive vascular and cellular changes. All slides were evaluated independently by two observers blinded to each other’s scores during the initial assessment. Complete blinding to clinical severity, MRI category, and anatomical region was not feasible, as the evaluation was performed in the context of an integrated imaging–histopathology correlation. In cases of discrepancy, a consensus was reached through joint re-evaluation. Semi-quantitative histopathological scores were assigned retrospectively, based on predefined evaluation criteria established prior to data analysis (Table S5). This approach was used to ensure consistency and reduce observer-related bias. To enhance methodological transparency and reproducibility, the complete case-level semi-quantitative scoring dataset, including individual scores and associated clinical and imaging data, is provided in the Supplementary Materials.

## 3. Results

### 3.1. Suspensory Ligament (Branch Region)

A total of 27 horses diagnosed with suspensory ligament injury were included in the study. The cohort comprised horses undergoing clinical, ultrasonographic, MRI, and histopathological evaluation. Cases represented a range of lesion severities, with clinical lameness graded according to the AAEP scale. Both branch-region and proximal-region lesions were represented in the analyzed material.

The distribution of MRI lesion categories included 10/27 (37.0%) category 1 lesions, 9/27 (33.3%) category 2 lesions, and 7/27 (25.9%) category 3 lesions. Ultrasonographically, 17/27 (63.0%) lesions showed a focal pattern, whereas 9/27 (33.3%) were classified as diffuse.

Histopathological assessment demonstrated frequent tissue alterations across the cohort. Edema was identified in 25/27 horses (92.6%), vascular alterations in 24/27 (88.9%), and extracellular matrix disorganization in 26/27 (96.3%). Moderate-to-severe changes (score ≥ 2) were present in 63.0% of edema scores, 55.6% of vascular scores, and 70.4% of extracellular matrix scores.

These findings indicate that the study population encompassed a broad spectrum of clinically and structurally relevant suspensory ligament lesions, allowing comparative correlation between imaging and histopathological parameters.

#### 3.1.1. General Histoarchitecture Morphological Histoarchitecture

**Figure 2:**
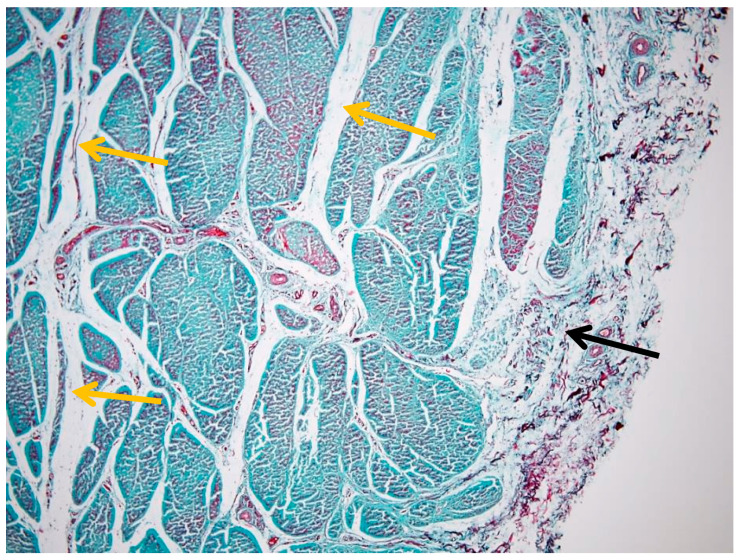
Overview of the suspensory ligament (Goldner’s trichrome stain, 4× objective) showing preserved histoarchitectural organization: black arrow—epitenon; yellow arrows—endotenon.

Transverse sections of the suspensory ligament reveal a well-defined histoarchitectural pattern, characterized by a hierarchical arrangement of connective tissue components. A distinct peripheral layer, the epitenon, surrounds the structure as a continuous sheath and exhibits a denser and more compact organization compared to the internal regions (Figure 2).

This layer is clearly demarcated from the underlying ligament substance. Connective tissue septa extend from the epitenon into the interior, forming an interconnected network that subdivides the structure into multiple fascicles. These septa, corresponding to the endotenon, delineate well-defined interfascicular boundaries and contribute to the lobulated appearance observed in transverse section (Figure 2).

Figure 2 illustrates the overall architecture, highlighting the distribution of polymorphic fascicles that vary in size and shape. These fascicles are composed predominantly of densely packed collagen fibers arranged in a parallel, slightly undulating pattern, while the interfascicular spaces appear less intensely stained due to the presence of looser connective tissue. Goldner’s trichrome staining clearly differentiates collagen-rich regions from the surrounding connective framework. Additional details of epitenon edema and early collagen fragmentation are provided in Figure S1, illustrating early structural alterations within otherwise preserved ligament architecture.

**Figure 3:**
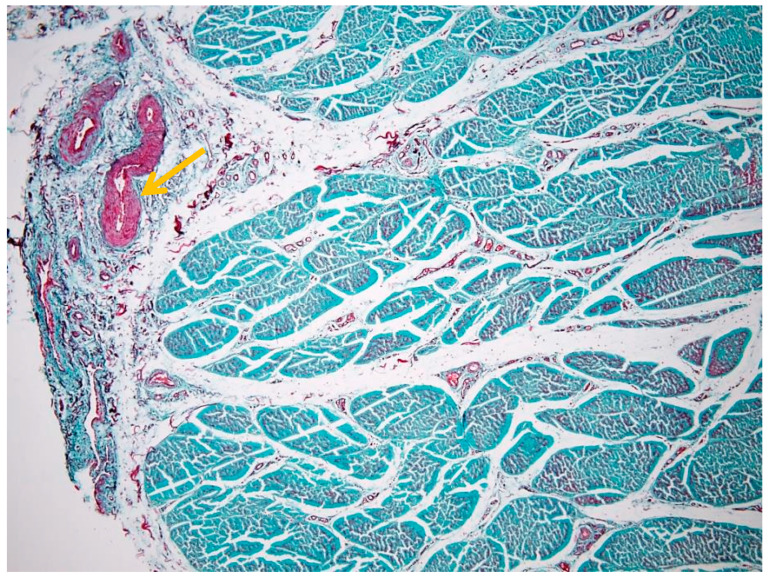
Interfascicular compartmentalization of the suspensory ligament (Goldner’s trichrome stain, 4× objective): the yellow arrow indicates the endotenon.

Figure 3 emphasizes the internal compartmentalization of the ligament, where the endotenon is more prominently visualized, separating collagen bundles into primary and secondary fascicles. These interfascicular septa contain vascular elements and connective tissue cells, contributing to tissue nutrition and structural maintenance.

The collagen bundles exhibit the characteristic greenish-blue staining associated with Goldner’s trichrome, reflecting their high collagen content and mechanical specialization. The epitenon (black arrow) is identifiable as the outer connective layer, while the endotenon (yellow arrow) forms the internal septal network. The peritenon, although not always distinctly visible at this magnification, facilitates gliding relative to adjacent structures.

Together, Figure 2 and Figure 3 provide complementary perspectives: Figure 2 defines the overall fascicular architecture, whereas Figure 3 highlights the interfascicular compartmentalization mediated by the endotenon.

#### 3.1.2. Vascular Distribution

**Figure 4:**
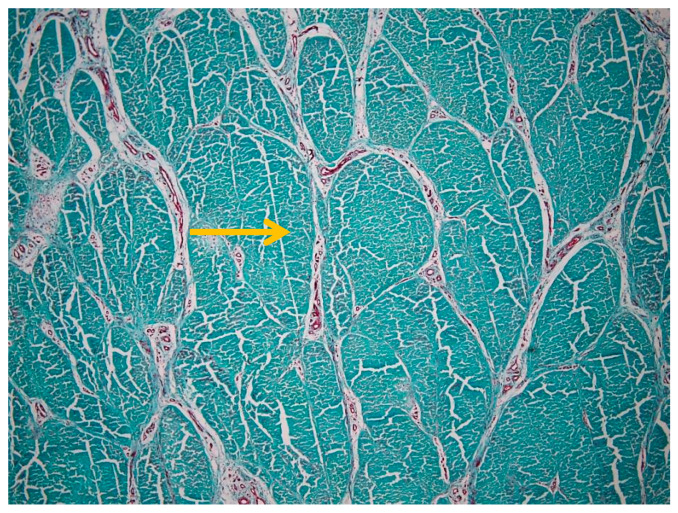
Vascular distribution within the peritenon (Goldner’s trichrome stain, 4× objective). The yellow arrow indicates tendon fascicles.

The histological image illustrates the distribution of blood vessels within the peritenon of the suspensory ligament, highlighted using Goldner’s trichrome staining at low magnification (4× objective) (Figure 4). The dense collagen bundles of the suspensory ligament are stained in greenish-blue, forming well-organized fascicles separated by connective tissue septa. Blood vessels were observed within the peritenon and interfascicular septa, forming an organized vascular network extending toward the internal compartments of the tendon (Figure 4). A more detailed overview of the vascular network within connective septa is shown in Figure S2.

**Figure 5:**
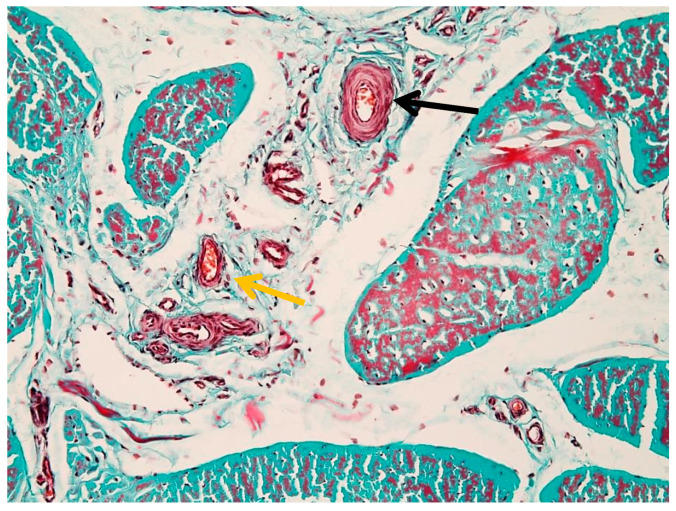
Vascular structures in the peritenon (Goldner’s trichrome stain, 20× objective): black arrow—arteriole; yellow arrow—venule.

Figure 5 illustrates the vascular component of the peritenon using Goldner’s trichrome staining at 20× magnification. The connective tissue matrix appears predominantly green–blue, consistent with collagen-rich structures, while vascular elements are stained in shades of red.

Multiple vessels of different calibers were identified within the peritenon, including arterioles and venules (Figure 5), with additional details shown in Figure S3. The arteriole (black arrow) is characterized by a thick wall and narrow lumen, while the venule (yellow arrow) shows a thinner wall and wider lumen.

#### 3.1.3. Edema

**Figure 6:**
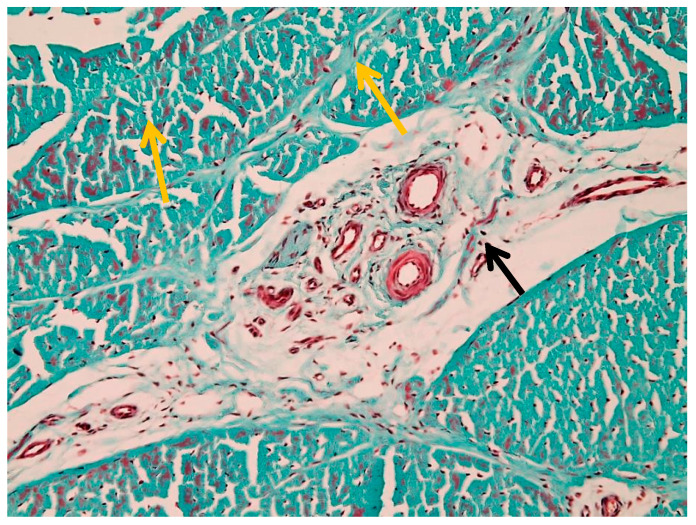
Generalized edema of the suspensory ligament (Goldner’s trichrome stain, 20× objective): black arrow—peritenon edema; yellow arrow—intrafascicular edema.

Figure 6 shows generalized edema involving the peritenon and intrafascicular matrix, with expansion and loosening of the peritenon. Collagen fibers are separated by optically clear spaces, while overall fascicular architecture remains preserved. Additional features, including intravascular thrombus formation, are shown in Figure S4.

#### 3.1.4. Perivascular Changes

**Figure 7:**
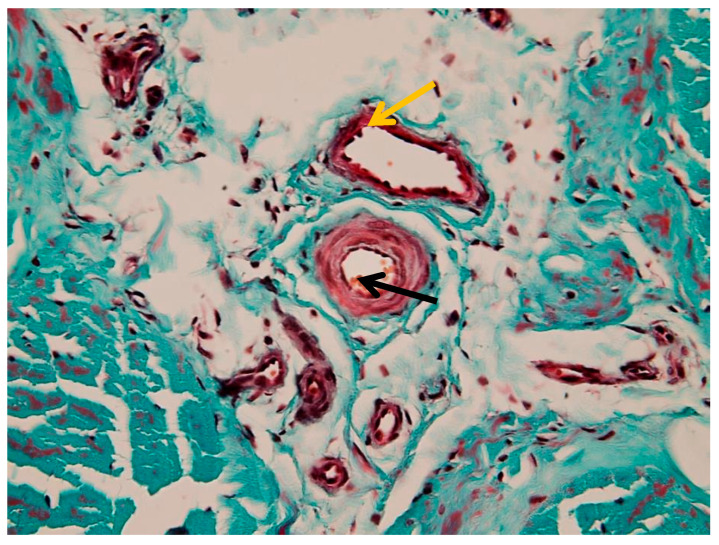
Marked perivascular edema associated with arterioles and venules in the peritenon (Goldner’s trichrome stain, 40× objective): black arrow—arteriole; yellow arrow—venule.

**Figure 8:**
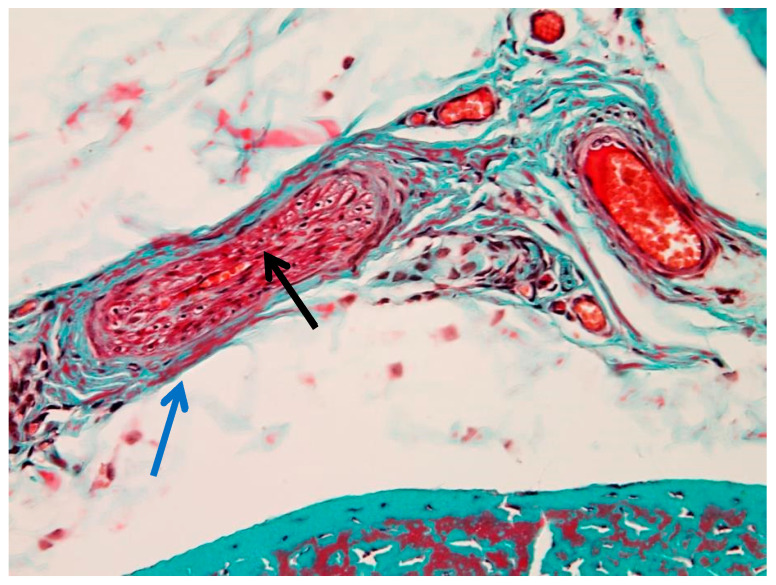
Perivascular alterations in the peritenon (Goldner’s trichrome stain, 40× objective): black arrow—apoptotic smooth muscle cells in the tunica media; blue arrow—pronounced perivascular edema.

Marked perivascular edema was observed, with expansion of the surrounding connective tissue and preserved vascular wall morphology (Figure 7). In more advanced areas, vascular wall degeneration and pronounced perivascular changes were present (Figure 8). Additional vascular alterations, including subendothelial connective tissue proliferation and perivascular edema, are illustrated in Figure S6. Advanced vascular remodeling with partial luminal narrowing and intraluminal erythrocyte accumulation is shown in Figure S7.

### 3.2. Suspensory Ligament (Proximal Region)

#### 3.2.1. General Histoarchitecture

**Figure 9:**
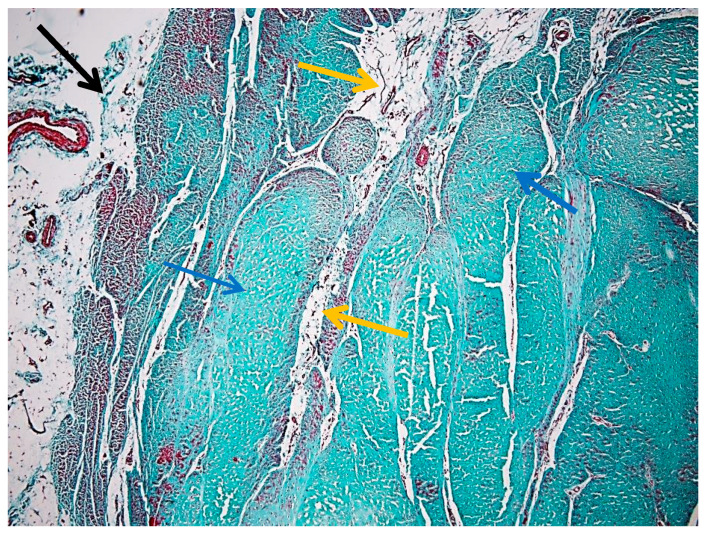
Overview of the suspensory ligament (Goldner’s trichrome stain, 4× objective) showing preserved histoarchitectural organization: black arrow—epitenon; yellow arrow—peritenon; blue arrow—tendon fascicles.

Figure 9 shows a low-magnification (4×) overview of tendon architecture stained with Goldner’s trichrome, which differentiates connective tissue components based on collagen content and organization. This image represents a region with relatively preserved histoarchitectural organization and is used as an internal reference for comparison with more structurally altered areas.

The suspensory ligament exhibits a hierarchical organization, from the outer connective tissue sheaths to the internal fascicular structure. The epitenon (black arrow) appears as a thin, dense connective tissue layer surrounding the tendon surface. The epitenon shows a compact and uniformly stained appearance. Surrounding and extending inward from the epitenon, the peritenon (yellow arrow) is visible as a looser connective tissue network. It presents a more irregular organization and reduced staining intensity compared to the epitenon. The bulk of the image is composed of tendon fascicles (blue arrow), arranged in parallel bundles of densely packed collagen fibers. These fascicles exhibit an elongated and wavy pattern, consistent with the typical crimped appearance of collagen fibers. The intense green–blue staining reflects the high collagen content.

**Figure 10:**
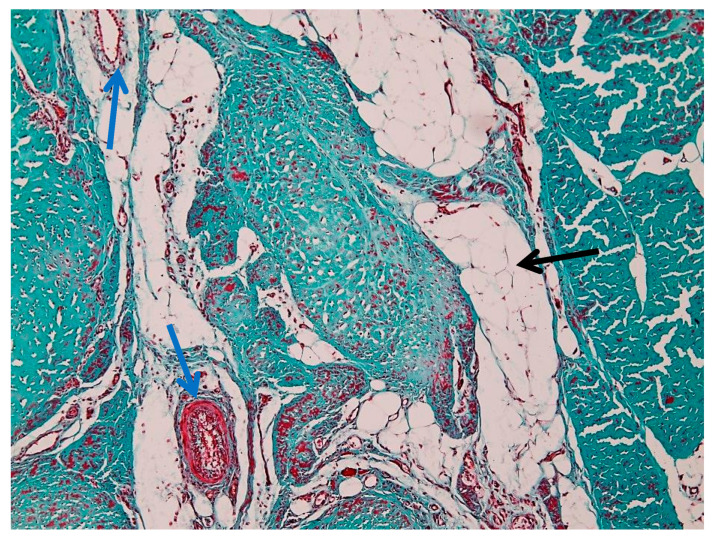
Zonal changes in the peritenon (Goldner’s trichrome stain, 10× objective): black arrow—adipose tissue; blue arrow—peritenon vessels.

Figure 10 shows zonal structural variations within the peritenon at intermediate magnification (10×), revealed by Goldner’s trichrome staining. The peritenon demonstrates a heterogeneous organization, with regions containing varying proportions of connective, vascular, and adipose components. Adipose tissue (black arrow) is observed as well-defined, optically clear vacuolated areas, while vascular structures (blue arrow) appear as luminal formations containing erythrocytes within the loose connective tissue matrix.

In addition to these components, the interfascicular compartment contained loose connective tissue, vascular elements, and adipocytes. In some sections, sparse muscle fibers were observed between fascicles, embedded within the interfascicular matrix [24]. These findings are consistent with previous descriptions of the suspensory ligament as a structure with mixed tendon–ligament–muscle characteristics; however, these fibers were limited to the interfascicular regions and did not contribute to the primary load-bearing collagen architecture [5].

This preserved structural organization serves as a reference framework for the interpretation of lesion-associated alterations observed in other regions, particularly in the context of extracellular matrix disorganization and vascular changes.

#### 3.2.2. Peritenon Characteristics

**Figure 11:**
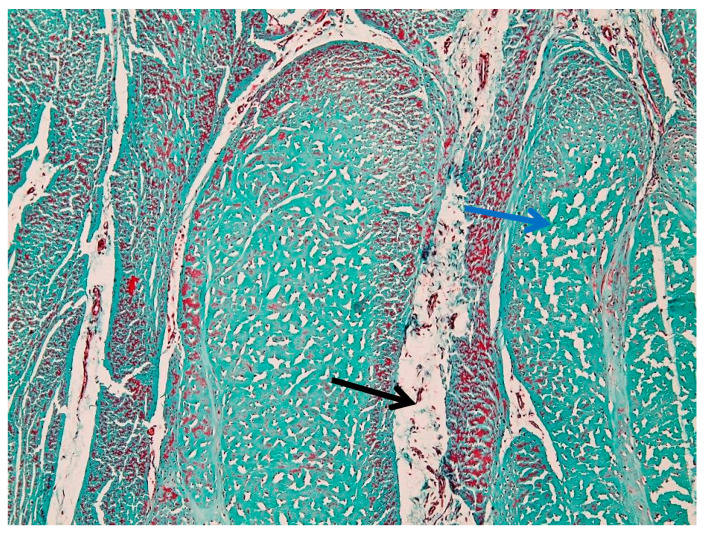
Generalized edema of the suspensory ligament (Goldner’s trichrome stain, 10×): black arrow—peritenon edema; blue arrow—intrafascicular edema.

**Figure 12:**
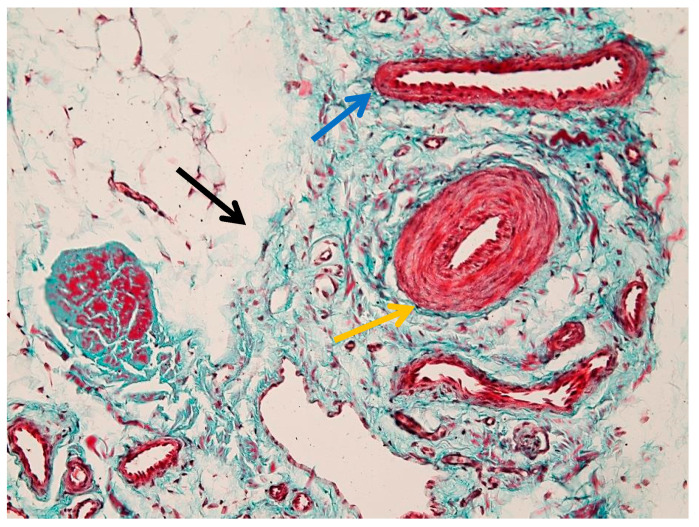
Perivascular edema in the epitenon (Goldner’s trichrome stain, 20×). Black arrow: perivascular edema; yellow arrow: artery; blue arrow: vein.

**Figure 13:**
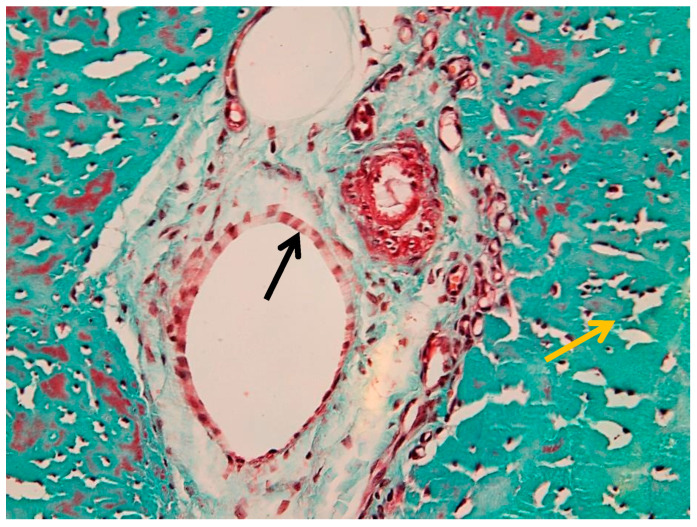
Endothelial and intrafascicular edema (Goldner’s trichrome stain, 40×). Black arrow: endothelial edema; yellow arrow: intrafascicular edema.

In injured suspensory ligament samples, diffuse edema involved both the peritenon and intrafascicular compartments, with relative preservation of the overall fascicular architecture (Figure 11). Perivascular and endothelial edema were also identified, characterized by enlargement of perivascular spaces and focal endothelial thickening (Figure 12 and Figure 13).

#### 3.2.3. Vascular Structural Alterations

**Figure 14:**
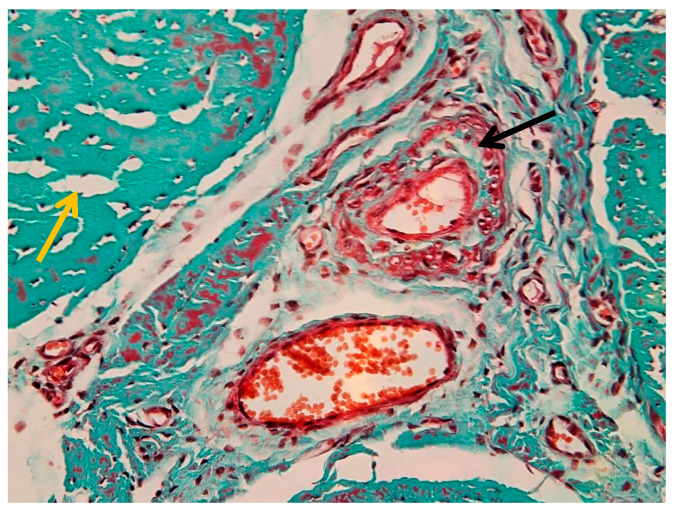
Perivascular connective tissue proliferation associated with intrafascicular edema (Goldner’s trichrome stain, 40×). Black arrow: connective tissue proliferation; yellow arrow: intrafascicular edema.

Figure 14 shows marked structural alterations of the vascular and perivascular connective tissue. The vessel wall appears thickened, with reduced definition of the normal vascular layers. The surrounding perivascular region is expanded by dense connective tissue, resulting in an irregular and disorganized architecture.

The adjacent extracellular matrix displays a heterogeneous appearance, with areas of increased density alternating with regions of rarefaction. Intrafascicular edema was present in adjacent tendon fascicles (Figure 14). The vascular lumen remains identifiable, with preserved intraluminal erythrocytes. The overall architecture is altered by expansion of the perivascular connective tissue and distortion of the surrounding ligament structure.

**Figure 15:**
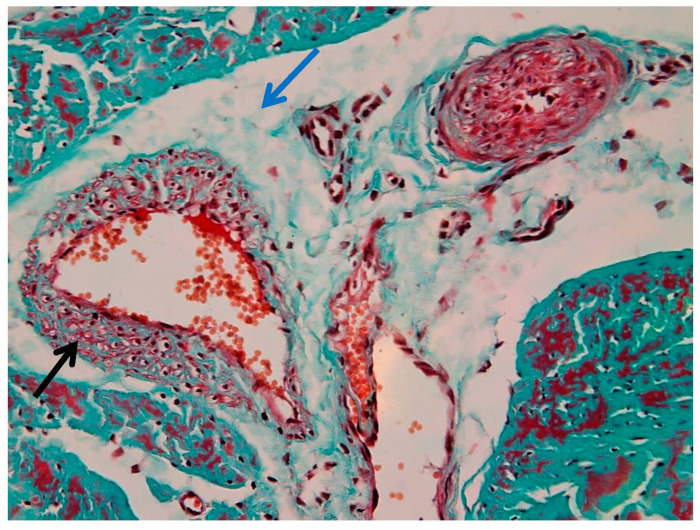
Medial fibrosis with associated perivascular edema (Goldner’s trichrome stain, 40×). Black arrow: medial fibrosis; blue arrow: perivascular edema.

Figure 15 shows vascular wall alterations characterized by marked thickening and increased density of the tunica media.

The medial layer appears more compact and homogeneous, with loss of the normal concentric organization. The perivascular space is expanded, containing loosely organized connective tissue with reduced staining intensity. Perivascular edema was also observed, associated with expansion of the surrounding connective tissue (Figure 15).

The adjacent connective tissue exhibits a heterogeneous structure, with areas of preserved organization alternating with regions of rarefaction. The vascular lumen remains identifiable and contains erythrocytes, while the overall vessel architecture is preserved despite the structural changes of the wall.

### 3.3. Comparative Histopathological Assessment

Both regions displayed a preserved baseline hierarchical organization, including identifiable epitenon, peritenon, and fascicular compartments, supported by a vascular network extending through the interfascicular connective tissue. However, the proximal region exhibited on average more extensive and structurally significant lesions than the branch region. In the branch region, changes were predominantly limited to perivascular and intrafascicular edema, with generally mild edema and limited structural alteration, although some branch cases showed severe lesions, including partial or complete rupture. The vascular architecture remained largely preserved. In contrast, the proximal region showed diffuse edema involving the epitenon, peritenon, and fascicular core, together with endothelial thickening, perivascular connective tissue expansion, vascular wall thickening, and, in selected vessels, medial fibrosis. Extracellular matrix alterations were more pronounced in this region, including collagen rarefaction, reduced matrix compactness, and structural remodeling. These findings indicate that the proximal region represents a higher-severity tissue involvement at the group level, rather than an absolute pattern, compared to the branch region (Table 1).

### 3.4. Lesion Distribution and Structural Variation Across Severity

Histopathological findings demonstrated a gradient of lesion severity, characterized by edema-dominated patterns and more extensive vascular and extracellular matrix alterations (Table 2). In the branch region, lesions were characterized by perivascular and intrafascicular edema with mild extracellular matrix expansion and limited collagen fiber separation, while overall tissue architecture remained preserved. In lesions with moderate severity, edema was more diffuse, involving the peritenon, epitenon, and deeper fascicular compartments, accompanied by endothelial thickening, perivascular connective tissue expansion, and moderate collagen disorganization. In the proximal region, lesions exhibited vascular wall remodeling, including wall thickening, medial alteration, and focal fibrosis, together with marked extracellular matrix expansion and collagen rarefaction.

These categories represent a conceptual classification of lesion severity based on cross-sectional observations and do not imply a temporal sequence or conceptual distribution of lesion severity.

**Figure 16:**
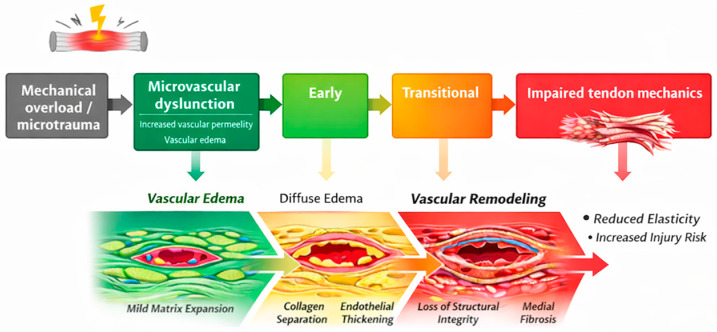
Distribution of histopathological changes across lesion severity patterns in the equine suspensory ligament.

These structural and vascular changes are schematically summarized in Figure 16. Additional representative histopathological findings supporting these lesion stages are provided in Figure S8.

### 3.5. Ultrasonographic Findings

Ultrasonographic examination identified two main lesion patterns: focal and diffuse. Focal lesions were characterized by localized hypoechoic or heterogeneous areas with disruption of the normal fibrillar architecture, whereas diffuse lesions showed more extensive alterations in echotexture, with reduced fiber alignment and no clearly demarcated defect.

As shown in Table 3, focal ultrasonographic patterns were observed in 17/27 cases (63.0%) and were associated with both MRI category 1 (10 cases, 37.0%) and MRI category 3 (7 cases, 25.9%), while no focal lesions were associated with MRI category 2. In contrast, diffuse ultrasonographic patterns were identified in 9/27 cases (33.3%) and were exclusively associated with MRI category 2 lesions (9 cases, 33.3%).

Case-level data (Table S6) confirmed this distribution, with all diffuse ultrasonographic patterns corresponding to remodeling or fibrotic MRI findings (category 2), whereas focal patterns were associated with either hyperintensity/edema (category 1) or partial/complete rupture (category 3).

This relationship was consistently reflected in the summarized distributions (Tables S7 and S8), which demonstrated that diffuse ultrasonographic categories corresponded exclusively to MRI category 2, while focal categories were distributed between MRI categories 1 and 3.

Overall, ultrasonographic findings showed a clear association with MRI-based lesion classification, with diffuse patterns corresponding to intermediate remodeling lesions and focal patterns encompassing both low- and high-severity structural changes. However, ultrasonography provided limited information regarding deeper structural and microvascular alterations compared to MRI.

### 3.6. Correlation Between MRI and Histopathological Findings

MRI findings showed a consistent correlation with the histopathological alterations observed in the suspensory ligament. MRI-based lesion categories showed distinct histopathological correlates. Category 1 lesions (edema/hyperintensity) corresponded to perivascular and intrafascicular edema with largely preserved ligament architecture. Category 2 lesions (remodeling/fibrosis) were associated with diffuse extracellular matrix disorganization, endothelial alterations, and perivascular connective tissue expansion. Category 3 lesions (partial or complete rupture) corresponded to marked collagen disruption, severe extracellular matrix disorganization, and advanced structural remodeling.

**Figure 17:**
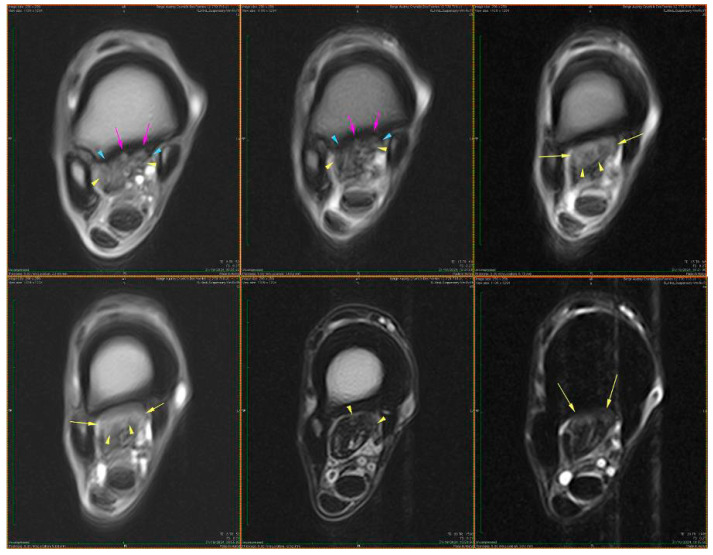
MRI sequences of the suspensory ligament: hyperintensity consistent with perivascular and intrafascicular edema, suggestive of microvascular dysfunction.

**Figure 18:**
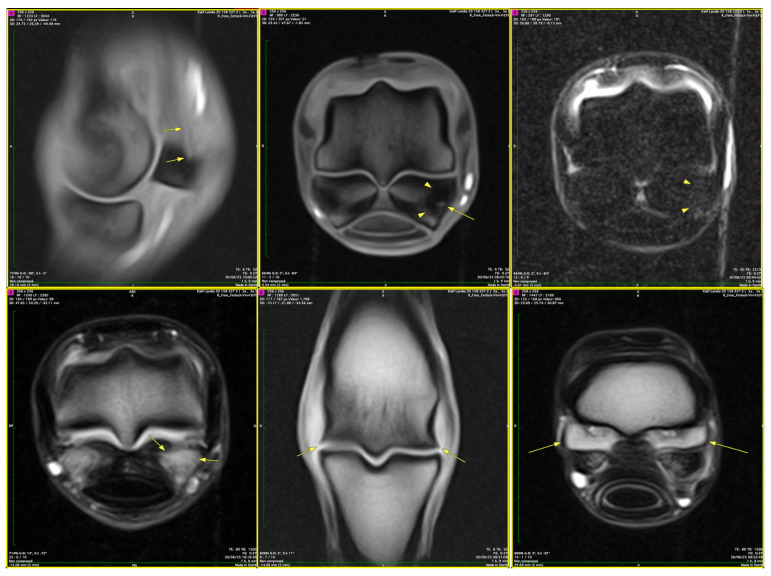
Multiplanar MRI sequences of the suspensory ligament (transverse and frontal views) showing areas of increased signal intensity consistent with perivascular and intrafascicular edema, associated with structural disorganization and ligament thickening.

**Figure 19:**
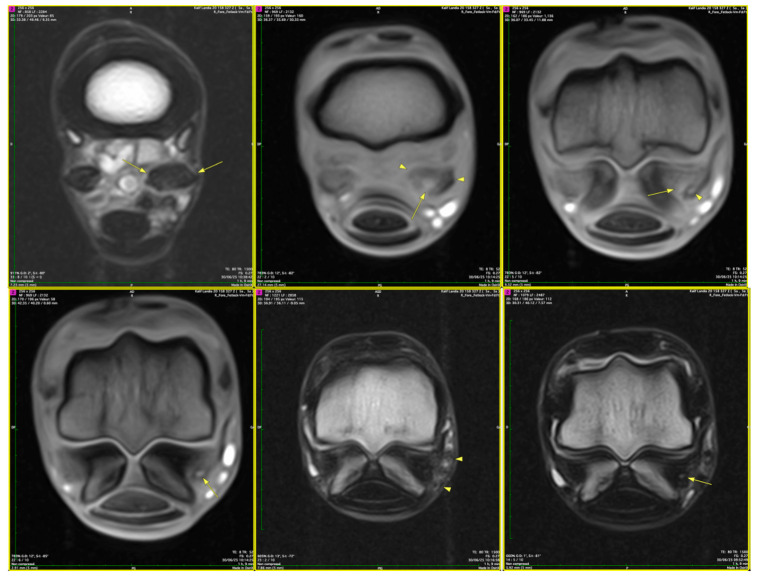
Transverse MRI sequences of the suspensory ligament showing focal and multifocal areas of increased signal intensity (arrows), consistent with perivascular and intrafascicular edema, associated with mild structural disorganization.

To further systematize the relationship between MRI signal alterations and their histopathological substrates, a structured imaging–histopathology correlation framework is presented (Table S9). Areas of increased signal intensity identified on fluid-sensitive sequences corresponded to regions of perivascular and intrafascicular edema demonstrated histologically (Figure 17, Figure 18 and Figure 19). In Figure 17, diffuse hyperintensity was evident, involving both perivascular and intrafascicular compartments. This pattern corresponded to widespread edema distribution, while the overall ligament architecture remained recognizable, without evidence of complete structural disruption.

In Figure 18, the signal alterations were more extensive and heterogeneous, with associated ligament thickening and contour irregularity. These imaging features were consistent with more advanced tissue involvement, characterized by increased structural disorganization of the ligament.

In Figure 19, focal and multifocal areas of hyperintensity were identified, corresponding to localized edema. In these regions, the overall fascicular architecture was preserved, consistent with lower-severity tissue involvement.

### 3.7. Quantitative Analysis and Statistical Correlations

The results presented in Table S10 demonstrate a coherent integration of clinical, imaging, and histopathological data across the 27 analyzed cases, enabling direct correlation between clinical severity (AAEP score), MRI findings, and microscopic alterations of the suspensory ligament. An increasingly pronounced distribution of histopathological scores (edema, vascular changes, and ECM alterations) can be observed in alignment with both clinical grade and imaging category, supporting a direct relationship between lesion severity and the extent of tissue disorganization. The final consensus score, established through independent evaluation and subsequent agreement between observers, strengthens the reliability of the dataset and provides a robust foundation for further comparative analyses. Histopathological changes were assessed using a semi-quantitative scoring system ranging from 0 (absent) to 3 (severe), based on structural, vascular, and extracellular matrix alterations.

Histopathological changes were distributed across the study population as follows: edema was observed in 25/27 horses (92.6%), with moderate-to-severe changes (score ≥ 2) in 17/27 cases (63.0%). Vascular alterations were identified in 24/27 horses (88.9%), with moderate-to-severe involvement in 15/27 cases (55.6%). Extracellular matrix (ECM) disorganization was present in 26/27 horses (96.3%), with moderate-to-severe changes in 19/27 cases (70.4%).

The results presented in Table 4, Table 5, Table 6, Table 7 and Table 8 provide strong and complementary evidence supporting the relevance and robustness of the study findings. Descriptive statistics (Table 4) show that most cases exhibit moderate to severe histopathological changes, with high proportions of scores ≥ 2 for edema (63.0%), vascular alterations (55.6%), and ECM changes (70.4%), indicating substantial tissue involvement. Edema was consistently localized within perivascular, interfascicular, and intrafascicular compartments, with more diffuse involvement observed in higher-severity lesions, particularly in the proximal region.

Correlation analysis (Table 5) demonstrates significant and clinically meaningful associations between MRI features and histopathology, with moderate to strong Spearman correlations (r_s_ = 0.52–0.68, *p* ≤ 0.006), confirming that imaging reliably reflects underlying microscopic pathology.

Interobserver agreement (Table 6) is high, with Cohen’s κ values ranging from 0.76 to 0.83 and agreement levels exceeding 80%, indicating excellent reproducibility of the scoring system.

Regional comparisons (Table 7) reveal significantly higher scores in proximal lesions compared to branch lesions (*p* = 0.002–0.006, moderate effect sizes), suggesting greater structural damage at the ligament origin.

Furthermore, clinical correlation (Table 8) shows that higher AAEP grades are significantly associated with increased histopathological severity (*p* < 0.05 across all parameters), reinforcing the link between microscopic damage and functional impairment. Clinical severity (AAEP grade) showed a clear association with lesion severity, with higher lameness grades corresponding to increased histopathological scores and more advanced MRI lesion categories. Overall, these findings collectively demonstrate that the dataset is highly relevant, internally consistent, and methodologically robust, providing a solid foundation for the study’s conclusions.

The observed patterns demonstrate variation in structural severity across samples; however, given the cross-sectional design of the study, no inference can be made regarding temporal sequence or causality. These findings should therefore be interpreted within a descriptive framework of lesion variability.

Despite the cross-sectional nature of the dataset, the consistent associations observed between histopathological parameters, imaging findings, and clinical severity allow the formulation of a structured model of lesion progression (Table S11). This model synthesizes the distribution patterns of edema, vascular alterations, and extracellular matrix changes, outlining a continuum from early perivascular edema and mild fiber separation, through intermediate diffuse vascular and structural disorganization, to advanced collagen rarefaction and late-stage irreversible remodeling. While this framework does not imply temporal causality, it provides a biologically plausible interpretation of lesion severity and supports the integrated imaging–histopathology correlations identified in the present study.

## 4. Discussion

### 4.1. Principal Findings and Interpretation

The present study demonstrates that suspensory ligament lesions are characterized not only by localized structural changes, but also by widespread alterations involving both the extracellular matrix and the microvascular compartment. To the best of our knowledge, this is the first study to provide an integrated histopathological characterization of microvascular involvement in the equine suspensory ligament, in direct correlation with MRI findings and regional lesion distribution.

The results of the present study support the concept that suspensory ligament lesions are not exclusively associated with mechanical factors, but involve a complex pattern of tissue alterations in which microvascular changes may play an important role. In contrast to earlier studies that predominantly emphasized structural degeneration and mechanical overload [20,23], our findings demonstrate a diffuse distribution of edema across perivascular, interfascicular, and intrafascicular compartments, suggesting a generalized disturbance of tissue homeostasis.

These observations are consistent with more recent research highlighting the involvement of the microvascular compartment in ligament pathology [5]. Furthermore, the observed association between increased vascularity/permeability and extracellular matrix disorganization suggests a potential interaction between vascular and structural components at the tissue level. However, given the cross-sectional design of the study, it is not possible to determine whether vascular alterations represent a primary event, nor whether edema constitutes the earliest lesion.

Therefore, the findings should be interpreted as demonstrating a correlation between imaging categories and semi-quantitative histological features, as well as differences between proximal and branch regions, rather than establishing a temporal or causal sequence of lesion development. The study was designed as a lesion-based comparative analysis, focusing on internal differences across regions and severity categories rather than direct comparison with healthy tissue. Therefore, the findings should be interpreted as reflecting relative variation within the spectrum of ligament injury [55].

Edema was consistently identified across perivascular, interfascicular, and intrafascicular regions, indicating that fluid accumulation is not restricted to vascular surroundings but affects the ligament architecture more broadly. In addition, more pronounced vascular alterations were observed in the proximal region compared to the branch region, suggesting a region-specific pattern of tissue remodeling.

Previous studies have primarily focused on structural degeneration and mechanical factors, whereas the present findings highlight the association between microvascular-related morphological alterations and lesion severity patterns. These findings support the hypothesis that vascular alterations may be associated with suspensory ligament injury, with links to increased vascular permeability, extracellular matrix disorganization, and greater structural compromise. However, alternative interpretations should be considered. The observed vascular and extracellular matrix changes may not exclusively reflect primary microvascular dysfunction, but could also represent secondary responses to mechanical overload or early degenerative processes. In this context, it remains difficult to determine whether vascular alterations are a driving factor or a consequence of tissue remodeling.

Taken together, these findings are consistent with regional differences reflecting variation in lesion severity, with more pronounced extracellular matrix disorganization observed in samples with higher severity scores.

Importantly, the present study is based on a cross-sectional design, and therefore the observed associations between vascular alterations, edema, and extracellular matrix changes should be interpreted as descriptive rather than mechanistic. The term microvascular involvement is used to describe morphological features observed at the tissue level, without implying direct functional impairment, causality, or temporal sequence.

### 4.2. Edema and Microvascular Dysfunction as Features of Lower Structural Severity

The presence of edema across multiple structural compartments indicates that fluid accumulation is a consistent feature observed in suspensory ligament lesions, particularly in lower-severity patterns. Rather than being confined to perivascular spaces, edema extended into interfascicular and intrafascicular regions, indicating a diffuse alteration of tissue homeostasis.

This pattern is compatible with alterations in the microvascular compartment, potentially involving increased vascular permeability and impaired regulation of fluid exchange. It should also be noted that similar patterns of edema may arise from inflammatory processes or transient vascular adaptations to mechanical stress. Therefore, the distinction between early pathological change and physiological adaptation remains uncertain, particularly in the absence of molecular or inflammatory markers. Despite these considerations, such alterations may be associated with the leakage of plasma components into the extracellular matrix, contributing to tissue swelling and local disruption of the microenvironment.

From a structural perspective, the accumulation of interstitial fluid can lead to separation of collagen fibers and disorganization of the extracellular matrix, thereby compromising the mechanical integrity of the ligament. These changes may impair the transmission of tensile forces and reduce the ability of the ligament to respond to repetitive loading.

Collectively, these findings suggest that lesions of lower structural severity are associated with a coupled pattern of microvascular-related changes and extracellular matrix destabilization, rather than isolated structural alterations.

### 4.3. Regional Differences and Distribution of Lesions Across Severity Categories

The present findings highlight clear regional differences between the branch and proximal portions of the suspensory ligament, indicating that lesions exhibit a non-uniform and spatially heterogeneous pattern. Although both regions exhibited edema and extracellular matrix alterations, the proximal region was characterized by more pronounced vascular changes, including endothelial alterations and perivascular matrix expansion, accompanied by greater structural disorganization. These features are consistent with a greater structural severity of tissue remodeling.

Furthermore, the comparative analysis between the proximal region and the ligament branches indicates region-specific differences in lesion severity, a feature only partially addressed in the previous literature. Earlier imaging and histological studies [6,8] have reported structural differences between these regions, but without explicitly integrating the vascular component. In contrast, our findings show that the proximal region exhibits more severe alterations, including vascular wall thickening, perivascular connective tissue proliferation, and tissue remodeling, compared to the more limited changes observed in the branch region. However, Supplementary Data (Tables S1 and S7) indicate that several branch cases are associated with partial or complete rupture and high histopathological scores, suggesting that severe lesions may also occur in branch regions. These findings should be interpreted as reflecting group-level differences in lesion characteristics and severity, rather than implying any ordered relationship between regions. The terminology used is intended to describe variation in structural features only.

Such regional disparities may reflect differences in biomechanical loading conditions, with the proximal portion likely subjected to increased and repetitive mechanical stress. This mechanical burden may exacerbate microvascular dysfunction and promote chronic tissue injury. In contrast, the branch region appears to represent a lower-severity pattern of tissue involvement, in which edema and mild extracellular matrix disruption predominate in the absence of significant vascular remodeling.

From a pathophysiological perspective, these observations support a pattern consistent with different levels of vascular alteration. In lower-severity lesions, a coordinated association between microvascular alterations and extracellular matrix instability was observed, accompanied by diffuse tissue changes. However, this interpretation should be approached with caution. The observed changes may also represent adaptive responses to altered mechanical loading rather than strictly pathological processes, particularly in lower-severity lesions. In these lower-severity patterns, the structural integrity of the ligament is relatively preserved, and the changes are likely functional and potentially reversible.

With increasing severity, persistent vascular alterations, including endothelial thickening and perivascular connective tissue expansion, are associated with increasing extracellular matrix disorganization. This is accompanied by collagen fiber separation, matrix rarefaction, and a reduction in tissue compactness, ultimately compromising the mechanical properties of the ligament. Continued exposure to mechanical stress further amplifies these changes, promoting advanced remodeling and structural deterioration.

**Figure 20:**
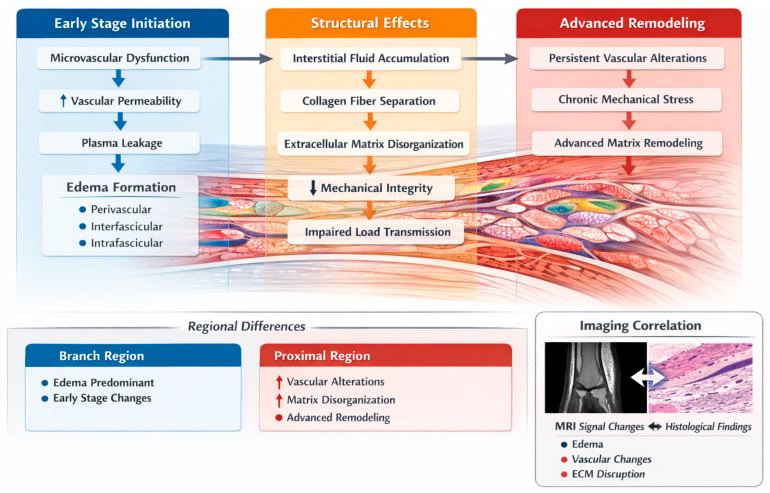
Conceptual model of lesion severity patterns in the equine suspensory ligament based on histopathological findings.

Within this framework, the proximal region may correspond to a higher-severity structural pattern, whereas the branch region reflects lower-severity alterations. This regional distribution is compatible with variation in structural severity across samples of tissue remodeling, rather than discrete pathological entities, and provides a more dynamic understanding of suspensory ligament pathology. Nevertheless, the proposed model should be interpreted with caution. Given the cross-sectional design of the study, causal relationships between the observed structural and vascular changes cannot be established, and no direct inference regarding temporal sequence can be made. Therefore, the proposed pattern of lesion distribution should be considered as a conceptual framework based on structural associations. Different regions may respond independently to local mechanical or vascular conditions, rather than representing a single uniform pathological process. Based on the observed histopathological features, a conceptual model of lesion severity in suspensory ligament lesions was proposed (Table S8), reflecting variation in lesion characteristics across samples. Building on these findings, a conceptual framework integrating microvascular dysfunction with extracellular matrix remodeling across increasing severity is illustrated in Figure 20.

This model provides a unifying framework linking lower-severity patterns with more pronounced structural changes and highlights the central role of the microvascular compartment in associated with lesion severity patterns.

These histopathological stages are further supported by MRI findings, where lower-severity lesions are characterized by focal or diffuse hyperintensity consistent with edema, while higher-severity categories show more heterogeneous signal patterns and structural alterations. This imaging–histology concordance supports the use of MRI as a tool for assessing lesion severity in vivo.

### 4.4. Imaging–Histology Correlation and Clinical Implications

The correlation between imaging findings and histological alterations represents a key aspect in understanding suspensory ligament pathology. The present study demonstrates that imaging abnormalities, particularly those detected by advanced modalities such as MRI, are supported by distinct microscopic changes, including edema, extracellular matrix disorganization, and vascular alterations.

The correlation between histopathological and imaging findings observed in this study further confirms previous reports regarding the utility of MRI in detecting early ligament lesions [13]. However, our data suggest that increased signal intensity on fluid-sensitive sequences reflects not only the presence of edema, but also underlying structural alterations involving the microvascular and extracellular matrix compartments and extracellular matrix remodeling.

In addition, in vitro evidence indicates that PRP and related biologics can influence the behavior of mesenchymal stem cells derived from equine synovial fluid, enhancing proliferation and modulating cellular responses [56]. However, the present study did not include therapeutic interventions, and therefore no direct conclusions regarding treatment efficacy can be drawn. These observations should be interpreted as contextual information rather than evidence supporting clinical application.

Although previous studies have reported the clinical use of platelet-rich plasma (PRP) and hyaluronic acid in equine musculoskeletal disorders [57,58], the present findings do not allow direct inference regarding therapeutic effectiveness. The observed association between edema, microvascular alterations, and extracellular matrix remodeling may, however, provide a pathophysiological framework for future studies. This may ultimately support more informed clinical decision-making and more accurate prognostic evaluation.

Thus, in agreement with more recent studies [15], MRI may be interpreted not only as a diagnostic tool, but also as a method for assessment of lesion severity, enabling differentiation between early potentially reversible changes and advanced structural remodeling.

In particular, the presence of edema observed histologically across multiple compartments provides a structural basis for signal changes detected on imaging, supporting the interpretation of these findings as indicators of active tissue pathology rather than incidental variations. Similarly, microvascular changes identified in the proximal region may contribute to imaging patterns that reflect ongoing remodeling processes. However, it should be acknowledged that imaging findings are not entirely specific, and similar signal alterations may arise from different underlying tissue processes, which may limit direct histological interpretation. Importantly, these findings suggest that imaging features, particularly those associated with edema, should not be interpreted as incidental findings, but rather as indicators of subtle but potentially clinically relevant tissue alterations. This has direct implications for diagnostic accuracy, as subtle imaging changes may reflect underlying microstructural damage even in the absence of overt structural disruption.

Clinically, this correlation is highly relevant, as it may help explain discrepancies between imaging findings and the severity of lameness. Subtle imaging changes may correspond to significant microscopic alterations, particularly in lower-severity lesions, whereas more pronounced structural remodeling may be associated with more evident imaging abnormalities.

These findings highlight the importance of integrating imaging data with an understanding of underlying histopathology to improve diagnostic accuracy and clinical decision-making. In this context, advanced imaging techniques such as MRI may provide valuable insights into lesion extent and stage, especially in cases where conventional imaging modalities fail to fully explain clinical signs. From a clinical perspective, this integrated imaging–histology approach may improve lesion characterization and staging, allowing for earlier detection and more precise assessment of variation in lesion severity. This may ultimately support more informed clinical decision-making, including targeted therapeutic strategies and more accurate prognostic evaluation.

Beyond simple correlation, MRI findings appear to reflect different patterns of tissue involvement, ranging from early edema-dominated changes to more advanced structural disorganization. In this context, MRI may provide a non-invasive means of assessing variation in lesion severity, allowing differentiation between potentially reversible early alterations and more advanced remodeling associated with structural compromise.

In addition to MRI, ultrasonographic findings demonstrated a consistent relationship with lesion patterns observed in the present study. Diffuse ultrasonographic patterns were exclusively associated with MRI category 2 lesions, corresponding to remodeling and fibrotic changes, whereas focal ultrasonographic patterns were associated with both low-severity signal alterations (MRI category 1) and high-grade structural disruption (MRI category 3). This distribution indicates that ultrasonography may reliably identify diffuse structural alterations associated with intermediate lesion severity, but has limited specificity in differentiating between early edema and advanced structural disruption within focal lesions. These findings support the role of ultrasonography as an initial diagnostic tool, while highlighting its limitations in precise lesion staging compared to MRI.

### 4.5. Limitations and Future Directions

This study has several limitations that should be considered when interpreting the findings, within the context of its comparative design. The study design is based on a structured comparative framework, in which regions exhibiting relatively preserved histoarchitectural organization were used as internal references, while comparisons between anatomical regions with different degrees of involvement enabled the identification of lesion-associated patterns.

Within this framework, histopathological findings are interpreted in relation to internal structural variation across lesion severity. Interpretation was further supported by established histological descriptions from the literature, as well as by the integration of clinical and imaging findings, strengthening the overall consistency and robustness of the results.

First, the absence of molecular and immunohistochemical analyses limits the ability to further characterize the biological mechanisms underlying the observed vascular and extracellular matrix alterations. In particular, the lack of specific markers restricts the differentiation between inflammatory, degenerative, and vascular-driven processes, as well as the precise identification of endothelial activation and cellular responses. Consequently, the interpretation of microvascular dysfunction remains primarily morphological and indirect, without molecular validation.

In addition, although a semi-quantitative histopathological scoring system was applied, the lack of fully standardized and externally validated criteria may introduce a degree of subjectivity in the assessment of tissue alterations.

Selection bias may be present due to the inclusion of cases with imaging-confirmed structural alterations, which may have led to an underrepresentation of very early or subclinical stages. As a result, the observed edema and microvascular changes should not be interpreted as representing the earliest events in disease development, but rather as features associated with relatively mild, yet already established, pathology. Accordingly, the findings should be interpreted within the context of a selected pathological population.

Furthermore, the heterogeneity of the study population, including variation in discipline, lesion chronicity, and individual biological response, may have contributed to variability in the observed histopathological and imaging findings, as these factors were not fully controlled or stratified.

An additional limitation is related to the exclusive use of post-mortem samples, which may introduce variability associated with tissue handling, ischemic changes, and post-mortem tissue alterations. Although standardized protocols were applied, these factors may have influenced the morphological characteristics observed.

Furthermore, MRI signal changes remain non-specific and may reflect a range of underlying processes, including edema, inflammation, or early degenerative changes, which limits precise tissue-level interpretation. In addition, the use of a low-field (0.27 T) MRI system may reduce sensitivity for detecting subtle lesions compared to high-field systems. Although a correlation between imaging and histopathology was observed, imaging findings cannot be considered fully specific for individual pathological mechanisms.

The present study design does not allow determination of temporal sequence or causality. Therefore, it cannot be established whether vascular alterations represent primary events or whether edema constitutes the earliest lesion. Instead, the findings should be interpreted as a cross-sectional characterization of lesion-associated patterns, demonstrating consistent associations between imaging features, histopathological alterations, and clinical severity, within a conceptual framework based on structural associations.

Future studies integrating longitudinal designs, as well as molecular and immunohistochemical approaches, would be essential to further characterize the biological mechanisms and improve the understanding of the underlying tissue alterations in suspensory ligament injury.

## 5. Conclusions

The findings of the present study indicate that microvascular alterations, edema, and extracellular matrix disorganization frequently coexist within the suspensory ligament and vary systematically across lesions of differing structural severity. Rather than demonstrating a defined pathogenetic sequence, these observations are more appropriately interpreted as being consistent with a severity gradient associated with microvascular alterations. Within this framework, lesions characterized by predominant edema and limited structural disruption are consistent with lower-severity patterns, whereas lesions exhibiting vascular remodeling and marked extracellular matrix disorganization correspond to higher-severity patterns. However, given the cross-sectional design of the study, no conclusions can be drawn regarding causality.

## Figures and Tables

**Table 1:** Comparative histopathological features of the suspensory ligament in the branch and proximal regions.

Histopathological Parameter	Branch Region	Proximal Region
Overall histoarchitecture	Preserved hierarchical organization (epitenon, peritenon, fascicles)	Preserved hierarchical organization
Vascular distribution	Organized vascular network within peritenon and endotenon	Comparable distribution, with associated structural alterations
Peritenon structure	Loose connective tissue with vascular components	Heterogeneous structure with matrix expansion
Edema distribution	Perivascular and intrafascicular	Perivascular, epitenon, peritenon, and intrafascicular (diffuse)
Extracellular matrix	Mild expansion	Marked expansion with reduced compactness
Collagen fiber organization	Mild separation	Marked separation and rarefaction
Perivascular changes	Edema with preserved vessel walls	Edema associated with perivascular matrix expansion
Endothelial alterations	Not observed or minimal	Endothelial thickening and luminal narrowing
Perivascular connective tissue	No significant proliferation	Prominent connective tissue proliferation
Vascular wall morphology	Preserved structure	Wall thickening with reduced layer definition
Medial fibrosis	Not identified	Present in selected vessels

**Table 2:** Distribution of histopathological features across lesion severity patterns.

Lesion Severity Pattern	Dominant Features	Vascular Status	Extracellular Matrix
Mild pattern (predominantly branch region)	Perivascular and intrafascicular edema	Increased permeability, preserved vessel walls	Mild matrix expansion, slight collagen separation
Moderate pattern	Diffuse edema (peritenon + fascicles)	Endothelial involvement	Moderate collagen disorganization and matrix expansion
Severe pattern (predominantly proximal region)	Diffuse edema + vascular remodeling	Endothelial thickening, vascular wall changes	Marked matrix expansion and collagen rarefaction
Marked remodeling pattern (focal areas)	Fibrosis and connective tissue proliferation	Structural vascular alteration (medial fibrosis)	Advanced matrix remodeling

**Table 3:** Relationship between ultrasonographic lesion patterns and MRI-based lesion classification.

Ultrasonographic Pattern	MRI Category 1	MRI Category 2	MRI Category 3	Total (*n*, %)
Focal lesions	10 (37.0%)	0 (0%)	7 (25.9%)	17 (63.0%)
Diffuse lesions	0 (0%)	9 (33.3%)	0 (0%)	9 (33.3%)
Total	10 (37.0%)	9 (33.3%)	7 (25.9%)	27 (100%)

**Table 4:** Descriptive statistics of histopathological scores.

Parameter	Score 0	Score 1	Score 2	Score 3	Mean ± SD	Median (IQR)	Min–Max	% ≥ Score 2
Edema	2	8	10	7	2.07 ± 0.85	2 (1–3)	0–3	63.0%
Vascular	3	9	9	6	1.89 ± 0.90	2 (1–3)	0–3	55.6%
ECM	1	7	11	8	2.19 ± 0.78	2 (2–3)	0–3	70.4%

**Table 5:** Correlation matrix between MRI and histopathology.

Histopathology	MRI Parameter	Spearman r_s_	95% CI	*p*-Value	Strength
Edema	STIR hyperintensity	0.68	0.42–0.84	<0.001	Strong
Edema	T2 signal increase	0.57	0.28–0.78	0.003	Moderate
Vascular	Ligament thickening	0.52	0.21–0.74	0.006	Moderate
ECM	Structural disorganization	0.61	0.33–0.80	0.002	Strong

**Table 6:** Interobserver agreement.

Parameter	Cohen’s κ	95% CI	% Agreement	Weighted κ	Interpretation
Edema	0.79	0.63–0.91	85%	0.81	Substantial–excellent
Vascular	0.83	0.68–0.93	88%	0.85	Excellent
ECM	0.76	0.59–0.89	82%	0.78	Substantial

**Table 7:** Regional comparison (Branch vs. Proximal).

Parameter	Branch (Mean ± SD)	Proximal (Mean ± SD)	Median Diff	*p*-Value	Effect Size (r)
Edema	1.72 ± 0.78	2.41 ± 0.69	+0.69	0.004	0.51
Vascular	1.58 ± 0.74	2.26 ± 0.81	+0.68	0.006	0.48
ECM	1.89 ± 0.71	2.48 ± 0.66	+0.59	0.002	0.56

**Table 8:** Clinical correlation (AAEP grade).

Parameter	AAEP 2–3 (Mean ± SD)	AAEP 4 (Mean ± SD)	*p*-Value
Edema	1.84 ± 0.72	2.36 ± 0.81	0.018
Vascular	1.65 ± 0.68	2.21 ± 0.77	0.012
ECM	1.97 ± 0.70	2.52 ± 0.65	0.009

## Data Availability

The data supporting the findings of this study are available from the corresponding author upon reasonable request.

## Supplementary Materials

The following supporting information can be downloaded at: https://www.mdpi.com/article/10.3390/ani16101432/s1, Figure S1. Histological aspect of epitenon edema with early collagen fragmentation (Goldner’s trichrome, 40×), illustrating mild structural alteration within a partially preserved ligament architecture. Figure S2. Histological Overview of Fascicular Tissue with Connective Septa and Vascular Network (Goldner’s Trichrome, 40×); Figure S3. Detail of Vascular Structures within Connective Septa Showing Congestion (Goldner’s Trichrome, Higher Magnification); Figure S4. Histopathological features of generalized edema in suspensory ligament (Goldner’s trichrome, 20×), showing peritenon and intrafascicular edema accompanied by intravascular thrombus formation; Figure S5. Suspensory ligament tissue showing preserved collagen fascicles separated by connective septa and associated vascular structures (Goldner’s trichrome, 20×); Figure S6. Histopathological vascular alterations (Goldner’s trichrome, 40×), showing subendothelial connective tissue proliferation associated with perivascular edema; Figure S7. Vascular remodeling with partial luminal narrowing and intraluminal erythrocyte accumulation (Goldner’s trichrome, 40×); Figure S8. Advanced vascular wall thickening with marked luminal deformation and near-obliteration (Goldner’s trichrome, 40×); Figure S9. Transverse ultrasonographic comparison of the suspensory ligament in a representative case; Figure S10. Transverse ultrasonographic comparison of the suspensory ligament in a representative case; Figure S11. Transverse ultrasonographic comparison of the suspensory ligament in a representative case; Table S1. Clinical, imaging, and histopathological characteristics of horses included in the study (*n* = 27); Table S2. Clinical, imaging, and histopathological characteristics of horses included in the study (*n* = 8); Table S3. MRI sequences and their diagnostic purpose in suspensory ligament evaluation; Table S4. Histopathological parameters and evaluation criteria; Table S5. Semi-quantitative histopathological scoring criteria; Table S6. Individual case data including ultrasonographic patterns, ultrasonographic categories, and corresponding MRI findings and categories; Table S7. Distribution of MRI categories according to ultrasonographic categories; Table S8. Distribution of MRI categories according to ultrasonographic pattern; Table S9. Imaging–histopathology correlation criteria; Table S10. Case-level semi-quantitative histopathological scoring dataset and imaging–clinical characteristics (*n* = 27); Table S11. Histopathological features across different severity grades of suspensory ligament lesions.

## Abbreviations

The following abbreviations are used in this manuscript:

SL	Suspensory Ligament
MRI	Magnetic Resonance Imaging
T1W	T1-Weighted
T2W	T2-Weighted
T2*W	T2-Star Weighted
GRE	Gradient Echo
FSE	Fast Spin Echo
STIR	Short Tau Inversion Recovery
H&E	Hematoxylin and Eosin
AAEP	American Association of Equine Practitioners
ECM	Extracellular Matrix

## References

[1] Dyson S. (1994). Proximal suspensory desmitis in the hindlimb: 42 cases. Br. Vet. J..

[2] Dyson S.J., Arthur R.M., Palmer S.E., Richardson D. (1995). Suspensory ligament desmitis. Vet. Clin. N. Am. Equine Pract..

[3] Dyson S. (2014). Hindlimb lameness associated with proximal suspensory desmopathy and injury of the accessory ligament of the suspensory ligament in five horses. Equine Vet. Educ..

[4] Williams M.R., Crisman E., Taylor B.M. (2024). Microvasculature of the suspensory ligament of the equine hind limb. Am. J. Vet. Res..

[5] Guest D.J., Birch H.L., Thorpe C.T. (2025). A review of the equine suspensory ligament: Injury prone yet understudied. Equine Vet. J..

[6] Schramme M., Josson A., Linder K. (2012). Characterization of the origin and body of the normal equine rear suspensory ligament using ultrasonography, magnetic resonance imaging, and histology. Vet. Radiol. Ultrasound.

[7] Gruyaert M., Pollard D., Dyson S.J. (2020). An investigation into the occurrence of, and risk factors for, concurrent suspensory ligament injuries in horses with hindlimb proximal suspensory desmopathy. Equine Vet. Educ..

[8] Werpy N.M., Denoix J.M. (2012). Imaging of the equine proximal suspensory ligament. Vet. Clin. N. Am. Equine Pract..

[9] Dyson S., Pollard D. (2024). Determination of Equine Behaviour in Subjectively Non-Lame Ridden Sports Horses and Comparison with Lame Sports Horses Evaluated at Competitions. Animals.

[10] Greve L., Dyson S.J. (2014). The interrelationship of lameness, saddle slip and back shape in the general sports horse population. Equine Vet. J..

[11] Rhodin M., Egenvall A., Andersen P.H., Pfau T. (2017). Head and pelvic movement asymmetries at trot in riding horses in training and perceived as free from lameness by the owner. PLoS ONE.

[12] Keegan K.G., Dent E.V., Wilson D.A., Janicek J., Kramer J., Lacarrubba A., Walsh D.M., Cassells M.W., Esther T.M., Schiltz P. (2010). Repeatability of subjective evaluation of lameness in horses. Equine Vet. J..

[13] Brokken M.T., Schneider R.K., Sampson S.N., Tucker R.L., Gavin P.R., Ho C.P. (2007). Magnetic resonance imaging features of proximal metacarpal and metatarsal injuries in the horse. Vet. Radiol. Ultrasound.

[14] Bischofberger A.S., Konar M., Ohlerth S., Geyer H., Lang J., Ueltschi G., Lischer C.J. (2006). Magnetic resonance imaging, ultrasonography and histology of the suspensory ligament origin: A comparative study of normal anatomy of warmblood horses. Equine Vet. J..

[15] van Veggel E.C.S., Vanderperren K., Selberg K.T., Bergman H.-J., Hoogelander B. (2024). The evolution of lesions on follow-up magnetic resonance imaging of the proximal metacarpal region in non-racing sport horses that returned to work (2015–2023). Animals.

[16] Murray R.C., Tranquille C.A., Walker V.A., Milmine R.C., Bak L., Tacey J.B., Bolas N.M. (2020). Magnetic resonance imaging findings in the proximal metacarpal region of 359 horses and proximal metatarsal region of 64 horses acquired under standing sedation. J. Equine Vet. Sci..

[17] Nagy A., Dyson S. (2012). Magnetic resonance imaging findings in the carpus and proximal metacarpal region of 50 lame horses. Equine Vet. J..

[18] Wilson A.M., McGuigan M.P., Su A., van den Bogert A.J. (2001). Horses damp the spring in their step. Nature.

[19] Thorpe C.T., Udeze C.P., Birch H.L., Clegg P.D., Screen H.R.C. (2012). Specialization of tendon mechanical properties results from interfascicular differences. J. R. Soc. Interface.

[20] Birch H.L., Bailey A.J., Goodship A.E. (1998). Macroscopic ‘degeneration’ of equine superficial digital flexor tendon is accompanied by a change in extracellular matrix composition. Equine Vet. J..

[21] Birch H.L. (2007). Tendon matrix composition and turnover in relation to functional requirements. Int. J. Exp. Pathol..

[22] Thorpe C.T., Clegg P.D., Birch H.L. (2010). A review of tendon injury: Why is the equine superficial digital flexor tendon most at risk?. Equine Vet. J..

[23] Patterson-Kane J.C., Firth E.C. (2009). The pathobiology of exercise-induced superficial digital flexor tendon injury in Thoroughbred racehorses. Vet. J..

[24] Thorpe C.T., Godinho M.S.C., Riley G.P., Birch H.L., Clegg P.D., Screen H.R.C. (2015). The interfascicular matrix enables fascicle sliding and recovery in tendon, and behaves more elastically in energy storing tendons. J. Mech. Behav. Biomed. Mater..

[25] Kjaer M. (2004). Role of extracellular matrix in adaptation of tendon and skeletal muscle to mechanical loading. Physiol. Rev..

[26] Screen H.R.C., Berk D.E., Kadler K.E., Ramirez F., Young M.F. (2015). Tendon functional extracellular matrix. J. Orthop. Res..

[27] Wang J.H.-C. (2006). Mechanobiology of tendon. J. Biomech..

[28] Kjaer M., Langberg H., Heinemeier K., Bayer M.L., Hansen M., Holm L., Doessing S., Kongsgaard M., Krogsgaard M.R., Magnusson S.P. (2009). From mechanical loading to collagen synthesis, structural changes and function in human tendon. Scand. J. Med. Sci. Sports.

[29] Magnusson S.P., Langberg H., Kjaer M. (2010). The pathogenesis of tendinopathy: Balancing the response to loading. Nat. Rev. Rheumatol..

[30] Heinemeier K.M., Kjaer M. (2011). In vivo investigation of tendon responses to mechanical loading. J. Musculoskelet. Neuronal Interact..

[31] O’Brien C., Marr N., Thorpe C.T. (2021). Microdamage in the equine superficial digital flexor tendon. Equine Vet. J..

[32] Peffers M.J., Thorpe C.T., Collins J.A., Eong R., Wei T.K.J., Screen H.R.C., Clegg P.D. (2014). Proteomic analysis reveals age-related changes in tendon matrix composition, with age- and injury-specific matrix fragmentation. J. Biol. Chem..

[33] Ribbans W.J., September A.V., Collins M. (2022). Tendon and ligament genetics: How do they contribute to disease and injury? A narrative review. Life.

[34] Snedeker J.G., Foolen J. (2017). Tendon injury and repair—A perspective on the basic mechanisms of tendon disease and future clinical therapy. Acta Biomater..

[35] Sharma P., Maffulli N. (2008). Tendinopathy and tendon injury: The future. Disabil. Rehabil..

[36] Pollitt C.C. (2010). The anatomy and physiology of the suspensory apparatus of the distal phalanx. Vet. Clin. N. Am. Equine Pract..

[37] Zauscher J.M., Estrada R., Edinger J., Lischer C.J. (2013). The proximal aspect of the suspensory ligament in the horse: How precise are ultrasonographic measurements?. Equine Vet. J..

[38] Lavagnino M., Wall M.E., Little D., Banes A.J., Guilak F., Arnoczky S.P. (2015). Tendon mechanobiology: Current knowledge and future research opportunities. J. Orthop. Res..

[39] Mosaid S., Lee P., Jihad Y. (2025). Advances in Achilles tendon tissue engineering: Integrating cells, scaffolds, and mechanical loading for functional regeneration. Bioengineering.

[40] Ahmed I.M., Lagopoulos M., McConnell P., Soames R.W., Sefton G.K. (1998). Blood supply of the Achilles tendon. J. Orthop. Res..

[41] Pufe T., Petersen W.J., Mentlein R., Tillmann B.N. (2005). The role of vasculature and angiogenesis for the pathogenesis of degenerative tendons disease. Scand. J. Med. Sci. Sports.

[42] Riley G. (2004). The pathogenesis of tendinopathy. A molecular perspective. Rheumatology.

[43] Nourissat G., Berenbaum F., Duprez D. (2015). Tendon injury: From biology to tendon repair. Nat. Rev. Rheumatol..

[44] Hodgson R.J., O’Connor P.J., Grainger A.J. (2012). Tendon and ligament imaging. Br. J. Radiol..

[45] Khan K.M., Cook J.L., Bonar F., Harcourt P., Åström M. (1999). Histopathology of common tendinopathies. Update and implications for clinical management. Sports Med..

[46] Riley G.P. (2005). Gene expression and matrix turnover in overused and damaged tendons. Scand. J. Med. Sci. Sports.

[47] Docheva D., Müller S.A., Majewski M., Evans C.H. (2015). Biologics for tendon repair. Adv. Drug Deliv. Rev..

[48] Zhang S., Ju W., Chen X., Zhao Y., Feng L., Yin Z., Chen X. (2022). Hierarchical ultrastructure: An overview of what is known about tendons and future perspective for tendon engineering. Bioact. Mater..

[49] Cook J.L., Purdam C.R. (2009). Is tendon pathology a continuum? A pathology model to explain the clinical presentation of load-induced tendinopathy. Br. J. Sports Med..

[50] Khan K.M., Cook J.L., Kannus P., Maffulli N., Bonar S.F. (2002). Time to abandon the “tendinitis” myth: Painful, overuse tendon conditions have a non-inflammatory pathology. BMJ.

[51] Docking S.I., Ooi C.C., Connell D. (2015). Tendinopathy: Is Imaging Telling Us the Entire Story?. J. Orthop. Sports Phys. Ther..

[52] Jaworski Ł., Zabrzyńska M., Klimaszewska-Wiśniewska A., Zielińska W., Grzanka D., Gagat M. (2022). Advances in Microscopic Studies of Tendinopathy: Literature Review and Current Trends, with Special Reference to Neovascularization Process. J. Clin. Med..

[53] Rees J.D., Stride M., Scott A. (2014). Tendons—Time to revisit inflammation. Br. J. Sports Med..

[54] Abate M., Silbernagel K.G., Siljeholm C., Di Iorio A., De Amicis D., Salini V., Werner S., Paganelli R. (2009). Pathogenesis of tendinopathies: Inflammation or degeneration?. Arthritis Res. Ther..

[55] Daradics Z., Popescu M., Cătoi C., Mircean M.V., Macri A., Mîrza O., Szakacs A., Daina S., Fetea F., Lupșan A.F. (2025). Forage carbohydrate profiles and endocrine morphometric interactions in traditionally managed horses from Romania. Life.

[56] Bungărdean D., Pall E., Daradics Z., Popescu M., Tripon M.A., Lupșan A.F., Crecan C.M., Morar I.A., Nicolescu A., Marcus I. (2025). In vitro effects of PRP, ozonized PRP, hyaluronic acid, paracetamol, and polyacrylamide on equine synovial fluid-derived mesenchymal stem cells. Life.

[57] Bungărdean D., Pall E., Daradics Z., Popescu M., Tripon M.A., Lupșan A.F., Marcus I., Morar I.A., Bora F.D., Crecan C.M. (2025). Regenerative intra-articular therapy with PRP and hyaluronic acid in equine stifle lameness: Integration with controlled exercise. HVM Bioflux.

[58] Bungărdean D., Pall E., Daradics Z., Popescu M., Tripon M.A., Lupșan A.F., Marcus I., Morar I.A., Bora F.D., Crecan C.M. (2025). Regenerative intralesional therapy with platelet-rich plasma and hyaluronic acid for equine proximal suspensory desmitis: Clinical outcomes following integration with controlled exercise. HVM Bioflux.

