# Supplementary material for: Edema and Structural Remodeling in Equine Suspensory Ligament Injury: A Severity Gradient Associated with Microvascular Dysfunction
Source: Animals (Basel). 2026 May 8;16(10):1432. doi: 10.3390/ani16101432 (PMC13203378; doi:10.3390/ani16101432)
Supplement: Supplementary file 1 [file animals-16-01432-s001.zip › animals-4263743-supplementary.pdf]

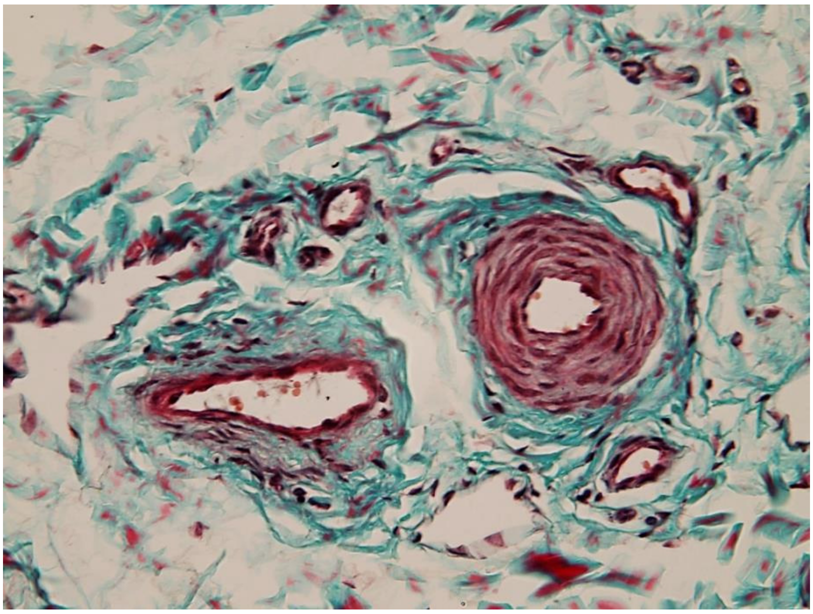

Figure S1. Histological aspect of epitenon edema with early collagen fragmentation (Goldner's trichrome, 40×), illustrating mild structural alteration within a partially preserved ligament architecture.

The figure illustrates a region of the suspensory ligament exhibiting epitenon edema and early collagen fragmentation, representing mild structural alteration within otherwise relatively preserved tissue. This staining technique allows clear differentiation of tissue components, with collagen fibers appearing in green shades and muscular and cellular structures in reddish tones. Within the microscopic field, an artery can be identified, recognized by its relatively round lumen and thick wall composed of concentric layers of smooth muscle. This structure is characteristic of arterial vessels, which regulate blood flow through contraction of the vascular wall. A vein is also present, characterized by a wider, more irregular lumen and a much thinner wall compared to the artery. This difference reflects the lower pressure in the venous system and the less robust structure of the venous wall. An important feature of the image is the presence of fragmented collagen fibers. These appear disorganized, interrupted, and dispersed within the extracellular matrix, indicating alteration of the typical connective tissue organization. Collagen fragmentation is a typical finding in the context of edema and tissue injury. Edema in the epitenon is manifested by the accumulation of fluid in the interstitial spaces, leading to separation and disorganization of collagen fibers. This explains the rarefied appearance of the connective tissue and the loss of its normal compact architecture.

This image illustrates early structural alterations within the epitenon, in the context of an otherwise preserved ligament architecture, and serves as a reference for comparative interpretation across lesion severity.

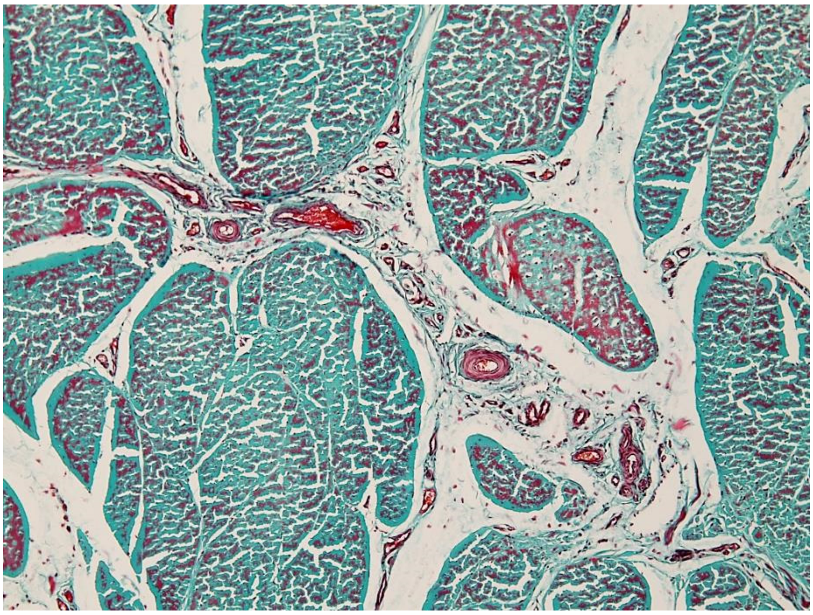

Figure S2. Histological Overview of Fascicular Tissue with Connective Septa and Vascular Network (Goldner's Trichrome, 40×)

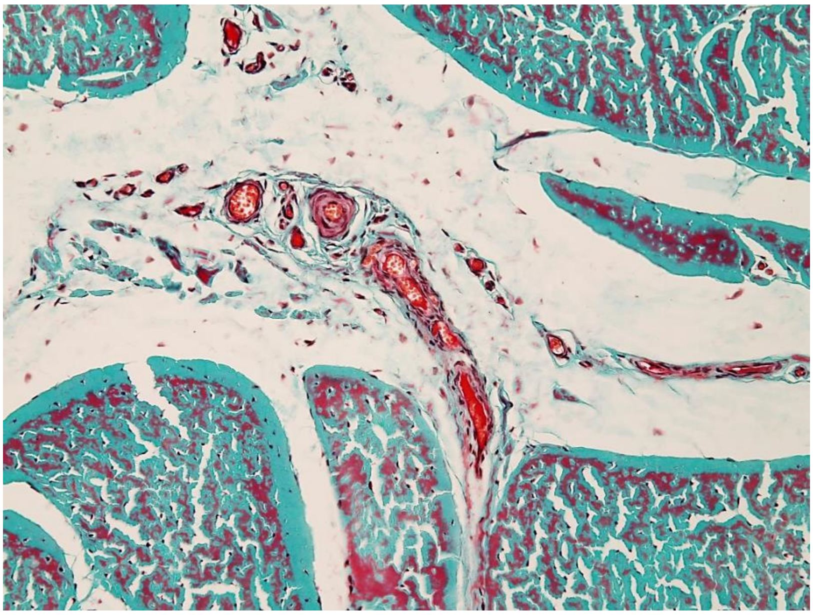

Figure S3. Detail of Vascular Structures within Connective Septa Showing Congestion (Goldner's Trichrome, Higher Magnification)

The histological examination of the tissue presented in Figures S2 and S3 reveals vascular and stromal alterations suggestive of early microcirculatory disturbances. In Figure S2, a well-organized fascicular architecture is evident, with connective tissue septa rich in collagen separating the fascicles and containing multiple blood vessels. Most vessels display relatively preserved morphology, with well-defined walls and patent lumina, indicating largely maintained vascular integrity.

Figure S3 provides a higher magnification view, highlighting vascular changes characterized by marked erythrocyte accumulation within the lumina, consistent with vascular congestion. In some vessels, the intraluminal content appears densely packed,

suggesting blood stasis and possible early erythrocyte aggregation rather than definitive intravascular coagulation. No clear evidence of organized thrombus formation or severe structural damage to the vascular wall is observed.

Additionally, the surrounding connective tissue shows mild loosening and separation of collagen fibers, which may reflect early interstitial fluid accumulation. These findings support the presence of localized microcirculatory impairment affecting both vascular and stromal compartments.

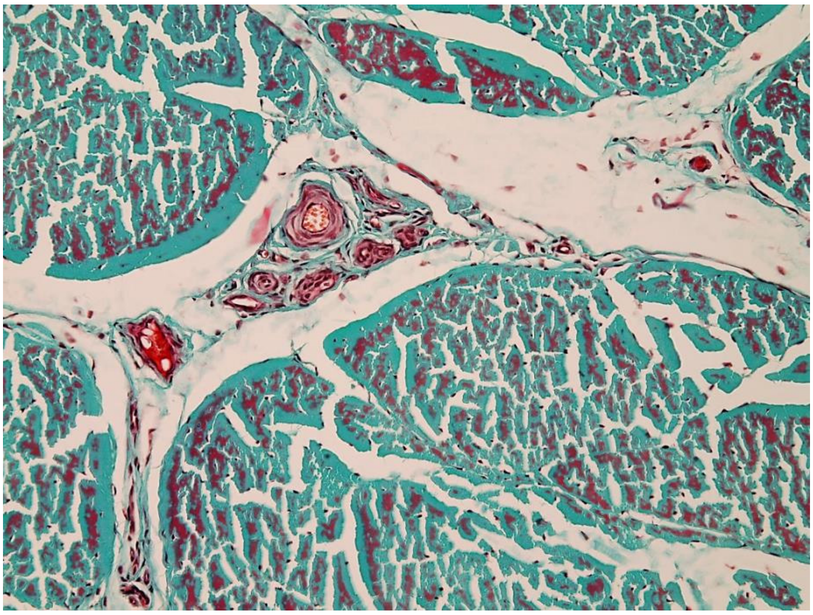

Figure S4. Histopathological features of generalized edema in suspensory ligament (Goldner's trichrome, 20×), showing peritenon and intrafascicular edema accompanied by intravascular thrombus formation.

The histological image (Goldner's trichrome stain, 20×) shows suspensory ligament tissue with a generalized edematous appearance. The normal compact organization of collagen fibers is disrupted by clear spaces, indicating fluid accumulation both around the fascicles (peritenon) and within them (intrafascicular edema). The connective tissue appears loosened and separated, with reduced density of collagen bundles. Additionally, vascular structures are visible, some containing dense intraluminal material consistent with intravascular coagulation (thrombus). Overall, the image reflects significant tissue alteration associated with edema and impaired microcirculation (Figure S4).

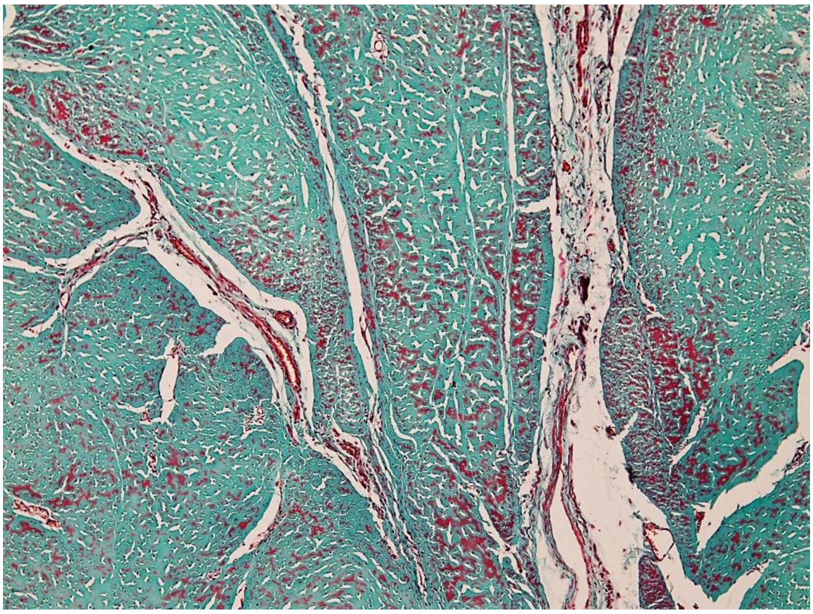

Figure S5. Suspensory ligament tissue showing preserved collagen fascicles separated by connective septa and associated vascular structures (Goldner's trichrome, 20×).

The histological image (Goldner's trichrome stain, 20×) shows suspensory ligament tissue with a well-preserved fascicular architecture. Collagen bundles are densely packed and organized in parallel arrays, separated by connective tissue septa. The interfascicular regions (endotenon) appear more loosely arranged and contain visible vascular structures, some filled with erythrocytes. The overall tissue organization is maintained, with no evidence of extensive structural disruption, although mild separation between fibers may suggest subtle interstitial fluid accumulation (Figure S5).

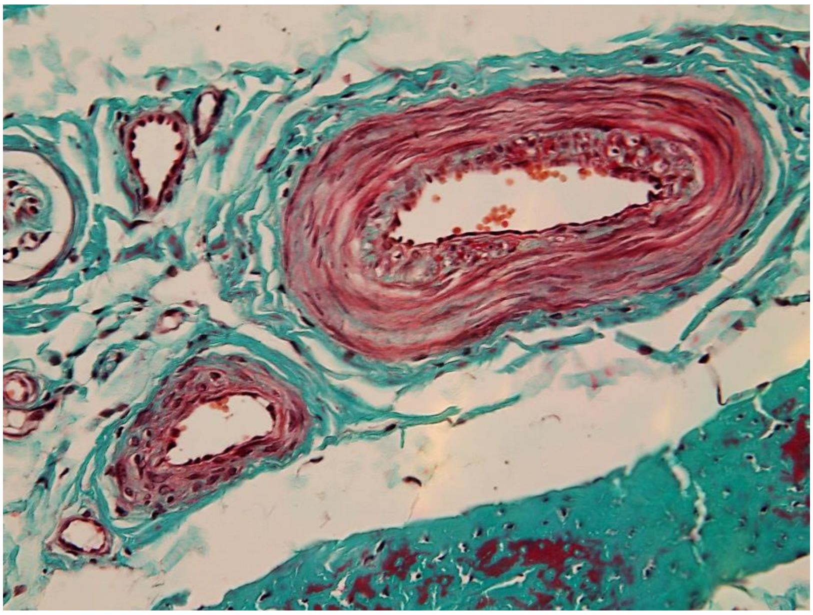

Figure S6. Histopathological vascular alterations (Goldner's trichrome, 40×), showing subendothelial connective tissue proliferation associated with perivascular edema

The histological image (Goldner's trichrome stain, 40×) shows vascular structures with marked wall thickening and structural remodeling. The vessel walls exhibit

prominent subendothelial connective tissue proliferation, resulting in narrowing of the vascular lumen. The surrounding perivascular tissue appears expanded and loosely organized, consistent with edema. Collagen fibers are highlighted in green, while the vascular smooth muscle and erythrocytes appear red. Overall, the image reflects significant vascular alteration associated with connective tissue proliferation and perivascular fluid accumulation (Figure S6).

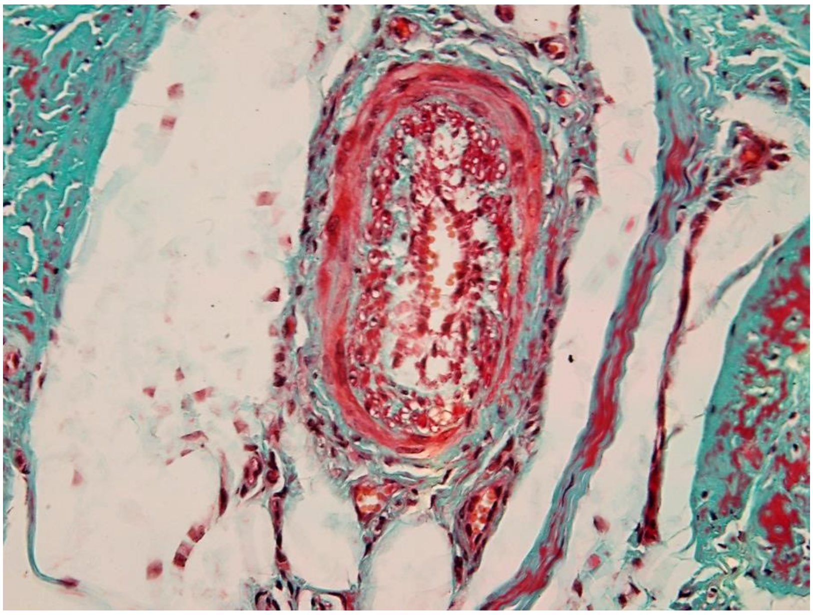

Figure S7. Vascular remodeling with partial luminal narrowing and intraluminal erythrocyte accumulation (Goldner's trichrome, 40×).

The histological image (Goldner's trichrome stain, 40×) shows a vessel with moderate wall thickening and partial luminal narrowing. The lumen contains accumulated erythrocytes, suggesting vascular stasis. The surrounding connective tissue appears mildly expanded, consistent with early perivascular edema. These findings are indicative of intermediate-stage vascular remodeling.

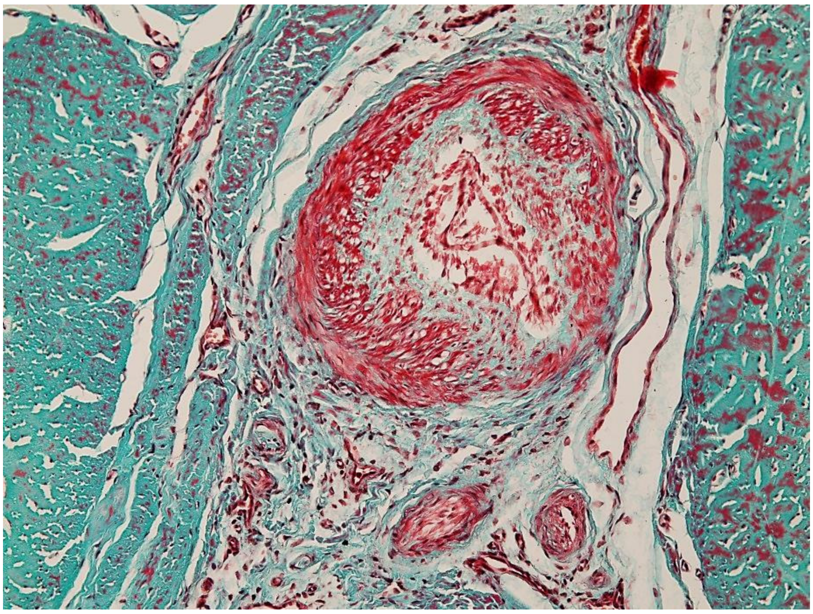

Figure S8. Advanced vascular wall thickening with marked luminal deformation and near-obliteration (Goldner's trichrome, 40×).

The histological image (Goldner's trichrome stain, 40×) shows severe vascular remodeling characterized by pronounced subendothelial connective tissue proliferation. This results in marked luminal deformation and near-complete obliteration. The surrounding connective tissue is expanded and loosely organized, consistent with perivascular edema. These findings reflect advanced-stage vascular alteration.

Table S1. Clinical, imaging, and histopathological characteristics of horses included in the study (n = 27)

ID	Age (years)	Sex	Breed	Discipline	Limb	AAEP grade	Duration	Ultrasound	MRI findings	Histological region	Notes
H01	9	M	Warmblood	Dressage	LF	3/5	chronic	focal hypoechoic	hyperintense	Proximal	—
H02	11	G	Warmblood	Jumping	RF	2/5	chronic	diffuse	remodeling	Proximal	—
H03	7	F	Thoroughbred	Racing	LF	3/5	acute	focal	rupture	Branch	—
H04	13	G	Warmblood	Dressage	RF	2/5	chronic	diffuse	fibrosis	Proximal	—
H05	10	M	Warmblood	Jumping	LF	3/5	chronic	focal	hyperintense	Branch	—
H06	8	F	Thoroughbred	Racing	RF	4/5	acute	focal	partial rupture	Branch	severe
H07	12	G	Warmblood	Dressage	LF	2/5	chronic	diffuse	remodeling	Proximal	—
H08	6	M	Thoroughbred	Racing	RF	3/5	acute	focal	edema	Branch	—
H09	14	G	Warmblood	Jumping	LF	2/5	chronic	diffuse	fibrosis	Proximal	—
H10	9	F	Warmblood	Dressage	RF	3/5	chronic	focal	hyperintense	Proximal	—
H11	7	M	Thoroughbred	Racing	LF	4/5	acute	focal	rupture	Branch	—
H12	11	G	Warmblood	Jumping	RF	2/5	chronic	diffuse	remodeling	Proximal	—
H13	10	F	Warmblood	Dressage	LF	3/5	chronic	focal	edema	Proximal	—
H14	8	M	Thoroughbred	Racing	RF	4/5	acute	focal	partial rupture	Branch	—
H15	12	G	Warmblood	Jumping	LF	2/5	chronic	diffuse	fibrosis	Proximal	—
H16	9	F	Warmblood	Dressage	RF	3/5	chronic	focal	hyperintense	Proximal	—
H17	6	M	Thoroughbred	Racing	LF	4/5	acute	focal	rupture	Branch	—
H18	13	G	Warmblood	Jumping	RF	2/5	chronic	diffuse	remodeling	Proximal	—
H19	10	F	Warmblood	Dressage	LF	3/5	chronic	focal	hyperintense	Proximal	—
H20	8	M	Warmblood	Jumping	RF	3/5	chronic	focal	edema	Proximal	—
H21	7	F	Thoroughbred	Racing	LF	4/5	acute	focal	rupture	Branch	—
H22	11	G	Warmblood	Dressage	RF	2/5	chronic	diffuse	fibrosis	Proximal	—
H23	9	M	Warmblood	Jumping	LF	3/5	chronic	focal	hyperintense	Proximal	—
H24	6	M	Thoroughbred	Racing	RF	4/5	acute	focal	partial rupture	Branch	—

H25	13	G	Warmblood	Dressage	LF	2/5	chronic	diffuse	remodeling	Proximal	—
H26	10	F	Warmblood	Jumping	RF	3/5	chronic	focal	hyperintense	Proximal	—
H27	10	F	Warmblood	Jumping	RF	3/5	chronic	focal	hyperintense	Proximal	—

Abbreviations: LF = left forelimb; RF = right forelimb; M = male (stallion); F = female (mare); G = gelding; AAEP = American Association of Equine Practitioners lameness grading scale (0–5). Age is reported in years at the time of clinical evaluation. Discipline refers to the primary athletic use of each horse (dressage, jumping, or racing). Limb indicates the affected forelimb based on clinical and diagnostic evaluation. AAEP grade represents the severity of lameness according to the standardized AAEP scale, where 0 = no lameness and 5 = non-weight-bearing lameness. Duration of clinical signs was classified as *acute* (<6 weeks) or *chronic* (>6 weeks), based on clinical history provided by the owner and referring veterinarian. Ultrasound findings describe echogenicity patterns within the suspensory ligament. *Focal hypoechoic lesions* indicate localized fiber disruption, whereas *diffuse changes* suggest generalized structural remodeling or chronic degeneration. MRI findings refer to signal intensity and structural abnormalities detected using magnetic resonance imaging. *Hyperintensity* is typically associated with edema or active inflammation, while *fibrosis* and *remodeling* indicate chronic degenerative changes. *Partial rupture* and *rupture* denote structural fiber disruption of varying severity. Histological region indicates the anatomical location of tissue sampling (proximal region or ligament branches), selected according to imaging findings to enable direct imaging–histopathology correlation. Notes include additional clinically relevant observations, such as lesion severity or suspected bilateral involvement.

Table S2. Clinical, imaging, and histopathological characteristics of horses included in the study (n = 8)

ID	Age (years)	Sex	Breed	Discipline	Limb	AAEP grade	Duration	Ultrasound	MRI findings	Histological region	Notes
E01	9	M	Warmblood	Dressage	LF	3/5	chronic	focal hypoechoic	not available	—	Excluded: MRI not available
E02	11	G	Warmblood	Jumping	RF	2/5	chronic	not available	remodeling suspected	—	Excluded: missing ultrasound data
E03	7	F	Thoroughbred	Racing	LF	3/5	acute	focal	poor image quality	—	Excluded: poor MRI quality
E04	10	M	Warmblood	Jumping	RF	2/5	chronic	diffuse	inconsistent findings	—	Excluded: lack of clinical–imaging concordance
E05	12	G	Warmblood	Dressage	LF	2/5	chronic	diffuse	no clear lesion	—	Excluded: no lesion identified on MRI
E06	8	F	Thoroughbred	Racing	RF	3/5	acute	focal	conflicting findings	—	Excluded: discordant imaging results
E07	13	G	Warmblood	Dressage	LF	2/5	chronic	diffuse	mild changes	—	Excluded: fetlock osteoarthritis

E08	9	M	Warmblood	Jumping	RF	3/5	chronic	focal	tendon abnormality	—	Excluded: concurrent SDFT injury

Abbreviations: LF = left forelimb; RF = right forelimb; M = male (stallion); F = female (mare); G = gelding; AAEP = American Association of Equine Practitioners lameness grading scale (0–5). Age is expressed in years at the time of clinical evaluation. Discipline refers to the primary athletic use of each horse (dressage, jumping, or racing). AAEP grade represents the severity of lameness, where 0 = no lameness and 5 = non-weight-bearing lameness. Duration of clinical signs was classified as *acute* (<6 weeks) or *chronic* (>6 weeks), based on clinical history. Ultrasound findings describe echogenicity patterns within the suspensory ligament. MRI findings refer to signal intensity and structural abnormalities detected by magnetic resonance imaging. Histological region is not applicable in excluded cases, as histopathological analysis was not performed. Horses were excluded due to incomplete imaging datasets, lack of concordance between clinical and imaging findings, or the presence of concurrent musculoskeletal disorders that could interfere with interpretation of suspensory ligament pathology. Reasons for exclusion are specified in the Notes column.

Table S3. MRI sequences and their diagnostic purpose in suspensory ligament evaluation

Sequence	Purpose
T1W GRE	Anatomical assessment
T2W FSE	Soft tissue evaluation
STIR	Fluid/edema detection
T2*W GRE	Trabecular bone assessment

Table S4. Histopathological parameters and evaluation criteria

Parameter	Description	Evaluation method
Collagen alignment	Degree of fiber organization and parallelism	Goldner trichrome
Fascicular structure	Integrity and continuity of fascicles	Goldner trichrome
Interfascicular spacing	Separation between collagen bundles	Goldner trichrome
Cellular morphology	Shape, density, and distribution of cells	Goldner trichrome
Vascular distribution	Density and spatial arrangement of vessels	Goldner trichrome
Edema	Interfibrillar expansion and fluid accumulation	Goldner trichrome

Table S5. Semi-quantitative histopathological scoring criteria

Score	Interpretation	Structural changes	Vascular changes	ECM changes
0	Normal	Normal architecture	Normal vessels	Normal matrix
1	Mild	Mild fiber disorganization	Mild vascular dilation	Mild ECM expansion
2	Moderate	Fascicular disruption	Endothelial alterations	Moderate ECM expansion
3	Severe	Loss of architecture	Wall thickening / fibrosis	Severe disorganization

Note: ECM = extracellular matrix. Scores range from 0 (normal) to 3 (severe).

Table S6. Individual case data including ultrasonographic patterns, ultrasonographic categories, and corresponding MRI findings and categories

ID	Ultrasound Pattern	Ultrasonographic Category	MRI Findings	MRI Category
H01	focal hypoechoic	Focal hypoechoic	hyperintense	1
H02	diffuse	Diffuse	remodeling	2
H03	focal	Focal	rupture	3
H04	diffuse	Diffuse	fibrosis	2
H05	focal	Focal	hyperintense	1
H06	focal	Focal	partial rupture	3
H07	diffuse	Diffuse	remodeling	2
H08	focal	Focal	edema	1
H09	diffuse	Diffuse	fibrosis	2
H10	focal	Focal	hyperintense	1
H11	focal	Focal	rupture	3
H12	diffuse	Diffuse	remodeling	2

H13	focal	Focal	edema	1
H14	focal	Focal	partial rupture	3
H15	diffuse	Diffuse	fibrosis	2
H16	focal	Focal	hyperintense	1
H17	focal	Focal	rupture	3
H18	diffuse	Diffuse	remodeling	2
H19	focal	Focal	hyperintense	1
H20	focal	Focal	edema	1
H21	focal	Focal	rupture	3
H22	diffuse	Diffuse	fibrosis	2
H23	focal	Focal	hyperintense	1
H24	focal	Focal	partial rupture	3
H25	diffuse	Diffuse	remodeling	2
H26	focal	Focal	hyperintense	1
H27	focal	Focal	hyperintense	1

Table S7. Distribution of MRI categories according to ultrasonographic categories

US Category	MRI 1	MRI 2	MRI 3	Total
Focal hypoechoic	1	0	0	1
Focal	10	0	7	17
Diffuse	0	9	0	9

Table S8. Distribution of MRI categories according to ultrasonographic pattern

US Pattern	MRI 1	MRI 2	MRI 3
Focal	10	0	7
Diffuse	0	9	0

Table S9. Imaging–histopathology correlation criteria

Imaging Finding	MRI Sequence	Corresponding Histopathology
Increased signal (STIR)	STIR	Edema
Decreased signal	T1W/T2W	Sclerosis
Ligament thickening	T2W/STIR	Fiber disorganization
Loss of structure	T2W	Fascicular disruption

Table S10. Case-level semi-quantitative histopathological scoring dataset and imaging–clinical characteristics (n = 27)

ID	Anatomical Region	AAEP Grade	MRI Category	Edema Score	Vascular Score	ECM Score	Observer 1	Observer 2	Final Consensus Score
H01	Proximal	3	1	1	1	1	1	1	1
H02	Proximal	2	2	1	2	2	1	2	2
H03	Branch	3	3	2	1	2	2	2	2
H04	Proximal	2	2	1	1	2	1	1	1
H05	Branch	3	1	1	1	1	1	1	1

H06	Branch	4	3	3	2	3	3	2	3
H07	Proximal	2	2	1	1	2	1	1	1
H08	Branch	3	1	2	1	1	2	1	2
H09	Proximal	2	2	1	2	2	1	2	2
H10	Proximal	3	1	2	2	2	2	2	2
H11	Branch	4	3	3	2	3	3	3	3
H12	Proximal	2	2	1	1	2	1	1	1
H13	Proximal	3	1	2	1	2	2	1	2
H14	Branch	4	3	3	3	3	3	3	3
H15	Proximal	2	2	1	2	2	1	2	2
H16	Proximal	3	1	2	2	2	2	2	2
H17	Branch	4	3	3	2	3	3	3	3
H18	Proximal	2	2	1	1	2	1	1	1
H19	Proximal	3	1	2	2	2	2	2	2
H20	Proximal	3	1	2	2	3	2	2	2
H21	Branch	4	3	3	3	3	3	3	3
H22	Proximal	2	2	1	1	2	1	1	1
H23	Proximal	3	1	2	2	2	2	2	2
H24	Branch	4	3	3	2	3	3	2	3
H25	Proximal	2	2	1	1	2	1	1	1
H26	Proximal	3	1	2	2	2	2	2	2
H27	Proximal	3	1	2	2	2	2	2	2

This table presents the case-level semi-quantitative histopathological scoring dataset for all included horses (n = 27), integrating clinical severity, imaging findings, and histopathological evaluation at the level of individual cases. Each row corresponds to a single subject, allowing direct correlation between clinical lameness grade, MRI characteristics, and microscopic structural alterations observed in the suspensory ligament. This integrated dataset provides the basis for subsequent descriptive and comparative analyses.

The anatomical region refers to the specific site of tissue sampling within the suspensory ligament, classified as either proximal (origin of the ligament) or branch (distal ligament branches). These regions were selected based on imaging findings in order to ensure accurate imaging–histopathology correlation and to allow comparative evaluation of lesion distribution across anatomically distinct compartments.

Clinical severity was assessed using the American Association of Equine Practitioners (AAEP) lameness grading scale. In the present dataset, only horses with clinically relevant lameness (grades 2 to 4) were included. Grade 2 corresponds to mild lameness, grade 3 to moderate lameness, and grade 4 to marked lameness. This grading system provides a standardized clinical framework for evaluating the relationship between microscopic changes and functional impairment.

MRI findings were categorized into three ordinal groups reflecting the predominant imaging pattern and presumed stage of lesion development. Category 1 includes edema or hyperintense signal changes, corresponding to fluid-dominant lesions typically associated with lower-severity lesion patterns. Category 2 includes remodeling or fibrosis, representing intermediate structural alterations characterized by matrix reorganization and partial loss of normal architecture. Category 3 includes partial rupture or complete rupture, reflecting advanced structural disruption with fiber discontinuity and severe tissue involvement. This classification was designed to reflect a continuum of lesion severity.

Histopathological evaluation was performed using a semi-quantitative scoring system applied to three key parameters: edema, vascular alterations, and extracellular matrix

(ECM) changes. Each parameter was scored on a scale from 0 to 3, where 0 indicates absence of detectable changes, 1 indicates mild alterations (such as minimal collagen separation or slight vascular dilation), 2 indicates moderate alterations (including evident fiber disruption, increased interfascicular spacing, and endothelial changes), and 3 indicates severe alterations characterized by marked structural disorganization, diffuse edema, vascular remodeling, and collagen rarefaction. Scoring was based on standardized morphological criteria, including collagen fiber organization, interfascicular architecture, vascular morphology, and matrix density.

Histopathological scoring was performed independently by two blinded observers in order to assess reproducibility and reduce subjective bias. Observer 1 and Observer 2 represent these independent evaluations. Minor discrepancies between observers are expected and reflect inherent variability in semi-quantitative interpretation of histological features.

The final consensus score represents the value used for statistical analysis and was established following joint re-evaluation of cases in which discrepancies between observers were identified. This consensus-based approach improves scoring reliability and ensures consistency across the dataset, providing a robust basis for subsequent quantitative analyses.

The dataset represents a structured semi-quantitative translation of histopathological findings, integrating clinical, imaging, and microscopic data within a unified analytical framework. Scores were assigned retrospectively based on predefined histopathological criteria and systematic interpretation of morphological features, rather than direct quantitative measurement. This approach is commonly applied in histopathological studies where fully quantitative measurements are not feasible, while still allowing reproducible and statistically analyzable data.

Table S11. Histopathological progression model of suspensory ligament lesions

Stage	Region	Edema	Vascular changes	Structural changes
Early	Branch	Perivascular	Increased vascular permeability	Mild fiber separation
Intermediate	Mixed	Diffuse	Endothelial thickening	Moderate fiber disorganization
Advanced	Proximal	Diffuse	Vascular wall thickening	Collagen rarefaction
Late	Focal	Variable	Fibrosis	Irreversible structural remodeling

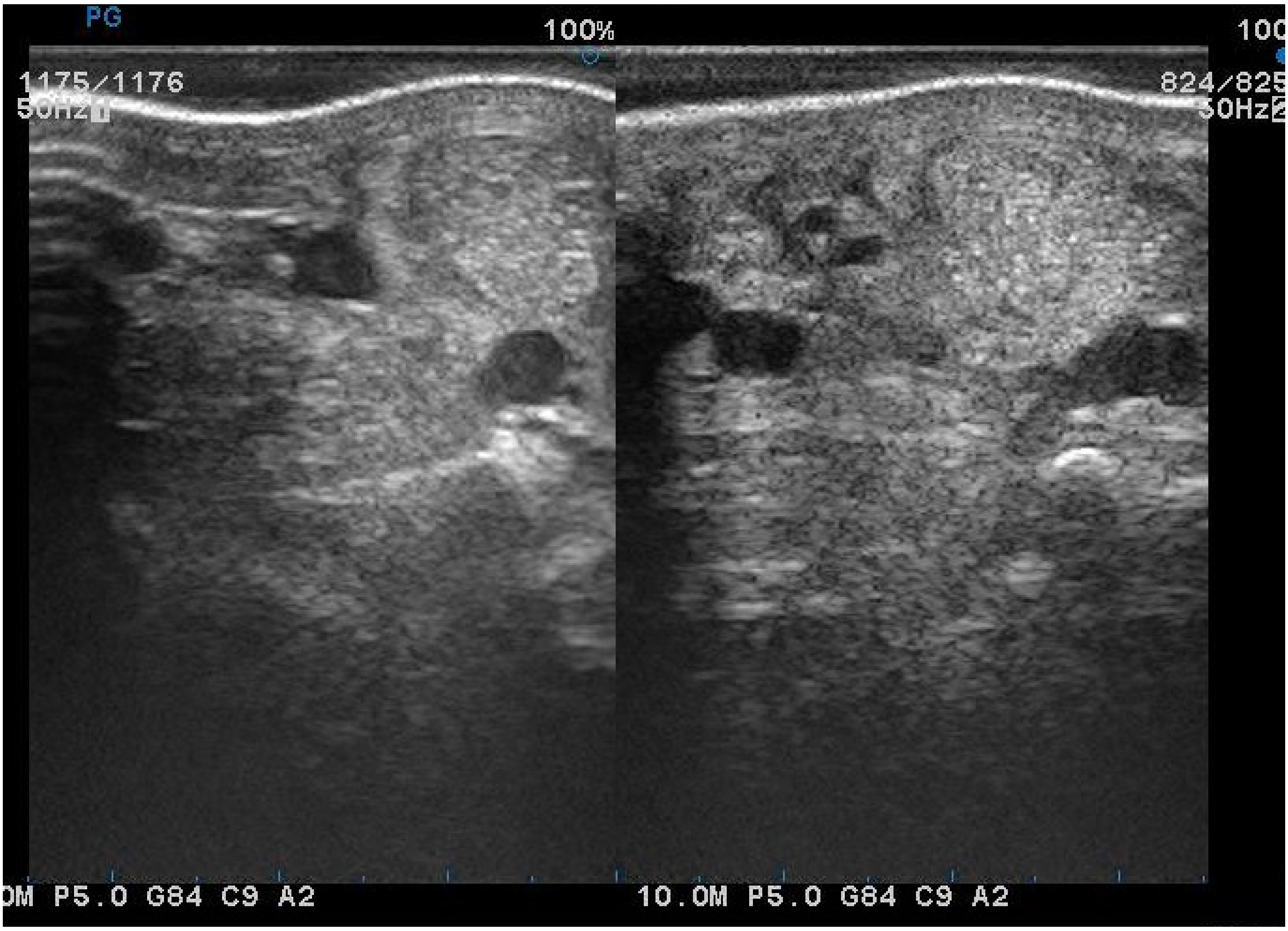

Figure S9. Transverse ultrasonographic comparison of the suspensory ligament in a representative case.

Right: affected limb demonstrating a focal hypoechoic defect and heterogeneous echotexture. Left: contralateral clinically normal limb with a more homogeneous and organized appearance. Images were obtained at the same anatomical level. The findings are consistent with lesion-associated changes when compared to the contralateral limb.

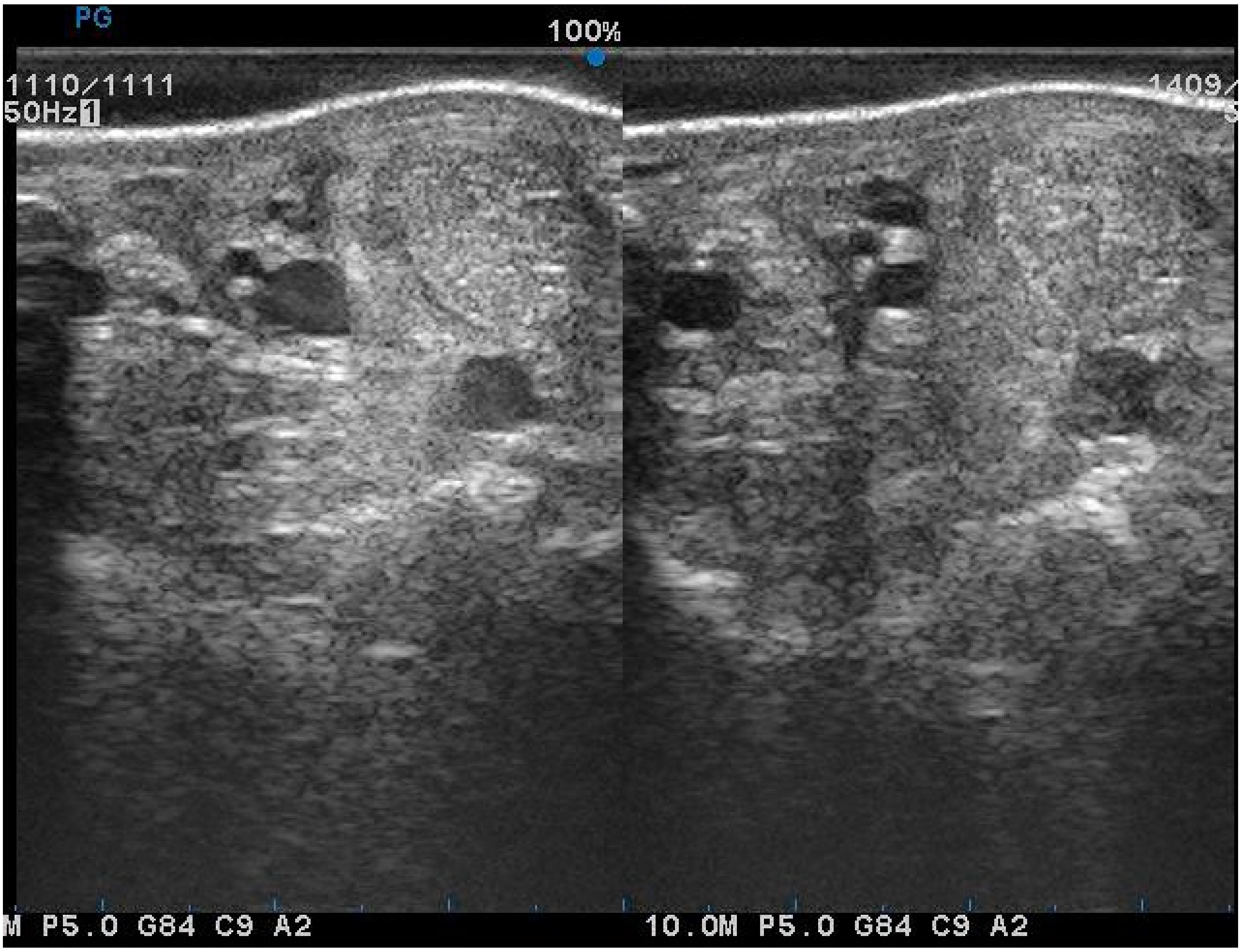

Figure S10. Transverse ultrasonographic comparison of the suspensory ligament in a representative case.

Right: affected limb demonstrating multiple hypoechoic areas and a heterogeneous echotexture, consistent with fiber disruption. Left: contralateral clinically normal limb with a relatively homogeneous and organized echotexture. Images were obtained at the same anatomical level. Findings are consistent with lesion-associated structural alterations when compared to the contralateral limb.

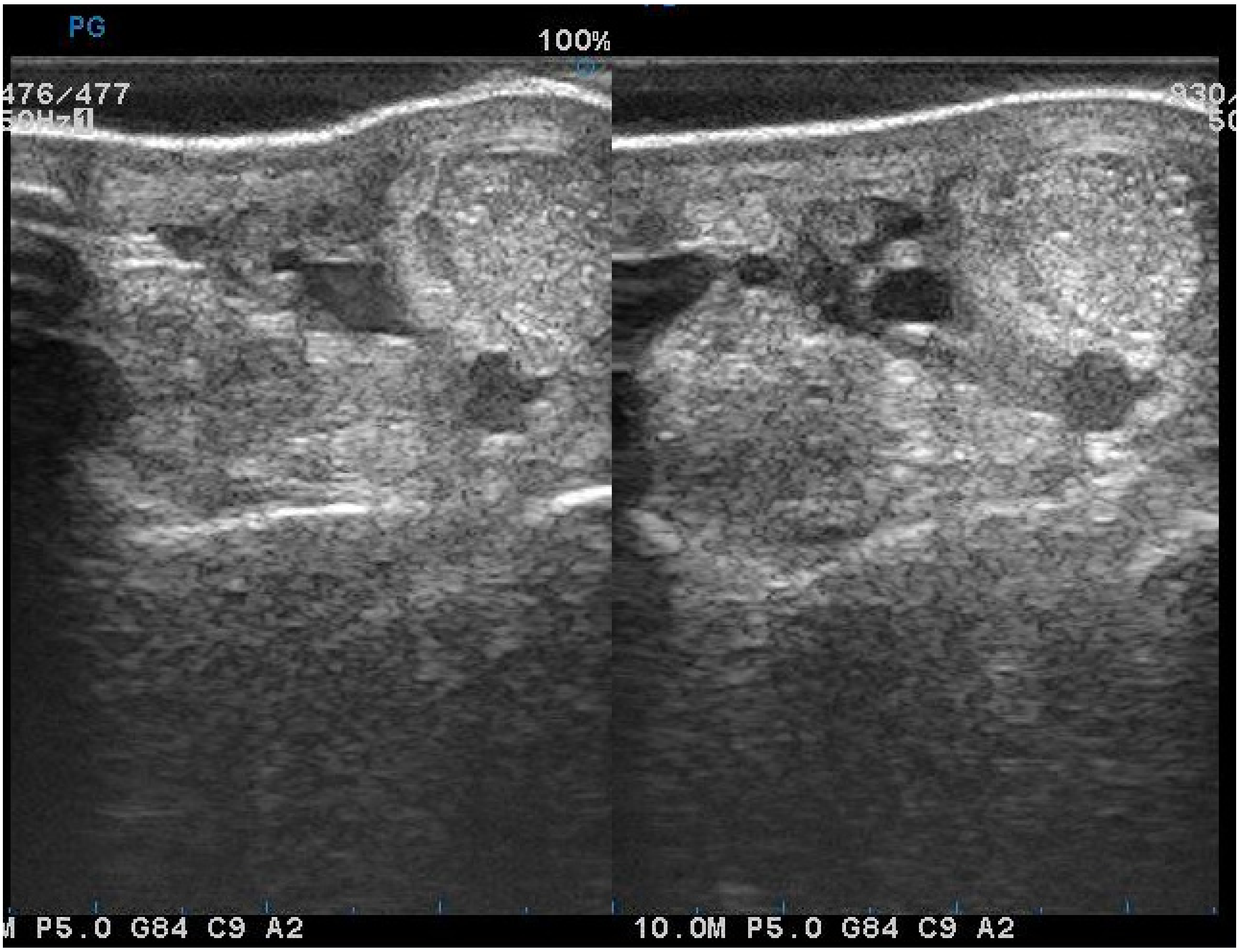

Figure S11. Transverse ultrasonographic comparison of the suspensory ligament in a representative case.

Right: affected limb demonstrating a focal to coalescing hypoechoic region with marked heterogeneity of the echotexture, suggestive of fiber disruption and structural disorganization. Left: contralateral clinically normal limb with a more uniform and organized echotexture. Images were obtained at the same anatomical level. Findings are consistent with lesion-associated changes when compared to the contralateral limb.
